# Extended methods for spatial cell classification with DBSCAN-CellX

**DOI:** 10.1038/s41598-023-45190-4

**Published:** 2023-11-01

**Authors:** Leonie Küchenhoff, Pascal Lukas, Camila Metz-Zumaran, Paul Rothhaar, Alessia Ruggieri, Volker Lohmann, Thomas Höfer, Megan L. Stanifer, Steeve Boulant, Soheil Rastgou Talemi, Frederik Graw

**Affiliations:** 1 BioQuant-Center for Quantitative Biology, Heidelberg University, 69120 Heidelberg, Germany; 2 Department of Molecular Genetics and Microbiology, College of Medicine, University of Florida, Gainesville, FL USA; 3 Department of Infectious Diseases, Virology, Center for Integrative Infectious Disease Research (CIID), Heidelberg University, 69120 Heidelberg, Germany; 4 Division of Theoretical Systems Biology, German Cancer Research Center (DKFZ), Heidelberg, Germany; 5 Interdisciplinary Center for Scientific Computing, Heidelberg University, 69120 Heidelberg, Germany; 6 Department of Medicine 5, Friedrich-Alexander-Universität Erlangen-Nürnberg, Schwabachanlage 12, 91054 Erlangen, Germany

**Keywords:** Cell biology, Computational biology and bioinformatics, Systems biology

## Abstract

Local cell densities and positioning within cellular monolayers and stratified epithelia have important implications for cell interactions and the functionality of various biological processes. To analyze the relationship between cell localization and tissue physiology, density-based clustering algorithms, such as DBSCAN, allow for a detailed characterization of the spatial distribution and positioning of individual cells. However, these methods rely on predefined parameters that influence the outcome of the analysis. With varying cell densities in cell cultures or tissues impacting cell sizes and, thus, cellular proximities, these parameters need to be carefully chosen. In addition, standard DBSCAN approaches generally come short in appropriately identifying individual cell positions. We therefore developed three extensions to the standard DBSCAN-algorithm that provide: (i) an automated parameter identification to reliably identify cell clusters, (ii) an improved identification of cluster edges; and (iii) an improved characterization of the relative positioning of cells within clusters. We apply our novel methods, which are provided as a user-friendly OpenSource-software package (DBSCAN-CellX), to cellular monolayers of different cell lines. Thereby, we show the importance of the developed extensions for the appropriate analysis of cell culture experiments to determine the relationship between cell localization and tissue physiology.

## Introduction

Human tissues are constituted by heterogeneous cellular structures that fulfill different functions. Besides their composition and the involved cell types that e.g. distinguish gut and respiratory epithelium, the spatial organization and positioning of cells in tissues has been intimately linked to their biological function^1^. Abnormal tissue architectures formed by the irregular spatial organization of cells in tissues are associated with different disease conditions and impaired tissue functionality^2^. Recent studies using novel methods of spatial-omics analyses and multiplexed imaging show gene expression differences in the vicinity of the pathogenic hallmarks in different tissues^3–5^. Thus, understanding the impact of the spatial organization of cells on tissue physiology and, thereby, on health or disease conditions is of utmost importance.

**Figure 1:**
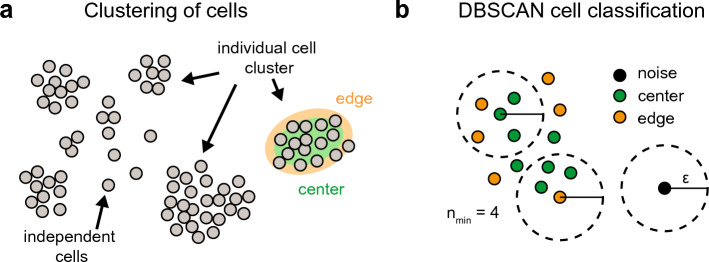
Clustering of cell populations: (**a**) Schematic of cell clustering with characterization of the location of individual cells. (**b**) Definition of the radius, $\varepsilon$, and the required minimum number of cells within the radius, $n_{{{\text{min}}}}$, to characterize cells as *center* (green), *edge* (orange) or *noise* cells (black) by DBSCAN.

To identify the location of different cells or cell states in a tissue or cell population, spatial clustering methods can be used to segregate cells into distinct subclasses—called cell clusters—where each cell cluster could consist of phenotypic similar, associated, or connected cells (Fig. 1a). Spatial statistics offers various methods to identify cellular clusters and cell clustering behavior, including supervised^6^ and unsupervised clustering methods, such as *k*-means clustering^7^ and model-based clustering^8,9^. Although many available clustering techniques can effectively accommodate the spatial relationships between cells, clustering cells given growth in arbitrary geometrical shapes, variable cellular densities, and typically large amounts of measurement noise, as well as characterizing the spatial context of individual cells, remains a challenge within biological research.

Among many clustering techniques available, density-based clustering methods are often used for analyzing biological/tissue samples^10^. Density-based clustering methods have relatively high scalability with respect to the number of clusters. The density-based spatial clustering of applications with noise (DBSCAN) is the best-known density-based clustering algorithm^11^. The DBSCAN algorithm involves a stepwise approach: in the first step, a spatial proximity relationship is built among the individual objects, in our case cells, by evaluating the local neighborhood of each object. If a sufficient number of other cells is found within the local surrounding of a cell, this cell is assumed to belong to a specific cluster. If not, the cell is defined as noise without contact to a cluster (Fig. 1b). DBSCAN not only assigns cells as belonging to a cluster or being noise, but also classifies them based on their local density within a cluster as being at the edge or densely packed within a cluster (Fig. 1b). To use DBSCAN, one has to define the size of the neighborhood and the minimum number of points in the neighborhood of each object to be considered as part of a cluster, making clustering sensitive to the choice of these parameters. Different methods have been developed to overcome this limitation and to improve the method's sensitivity to variation in the density of objects in space^12–15^. While these methods improve the identification of individual clusters, the classification of cells based on their location within clusters is still hampered by the standard classifications of DBSCAN and the generally irregular shapes and densities of patches within cell culture experiments.

Here, we propose DBSCAN-CellX as an extension to DBSCAN that provides a statistical identification of the DBSCAN parameters based on a generalized functional relationship between cell density and distribution, and especially aims at improving identification of cell positioning within clusters by a geometrical treatment of the DBSCAN prediction. Compared to DBSCAN, DBSCAN-CellX demonstrated better identification of local boundaries (i.e., edges) of cell clusters on simulated and experimental datasets. In addition, DBSCAN-CellX provides a method to classify cells based on their relative positioning within a cluster, which could help to improve the characterization of cellular functionality dependent on their local context. DBSCAN-CellX is provided as an OpenSource python package to analyze and classify microscopy images of cell populations that includes a user-friendly graphical user interface for non-expert users.

## Results

### Selecting appropriate parameters for density-based clustering of tissue cultures

**Figure 2:**
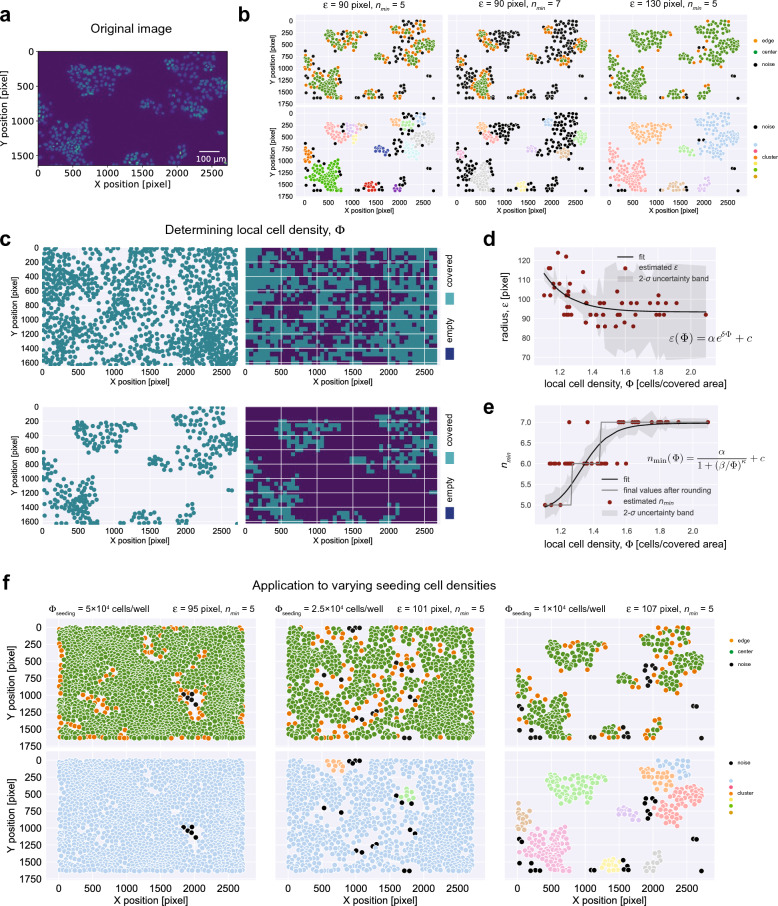
Identifying appropriate parameters for DBSCAN for cell culture data: (**a**) Original image and (**b**) example of cell characterization using DBSCAN with different combinations for the parameters $\varepsilon$ and $n_{{{\text{min}}}}$ based on images of T84 pMx1-mCherry H2B-turquoise cells grown at a cell seeding density of $\Phi_{{{\text{seeding}}}} = 10^{4}$ cells/well. (**c**) Calculation of the average local cell density $\Phi$ by separating images into regular grids of 20 μm grid size and classification of local areas as being covered or empty dependent on the presence or absence of cells within the particular grid sites, respectively. (**d,e**) Specification of the radius, $\varepsilon$, and the minimum number of cells, $n_{{{\text{min}}}}$, as functions of the average local cell density,$\Phi$. The manually characterized images (dots) and fitted functions (best fit—line, 95%-confidence interval—shaded area) are shown. For $n_{{{\text{min}}}} \left( \Phi \right)$ the continuous function, as well as the integer-valued step-function using rounded values is shown (see also Table 1). (**f**) Characterized images from different experiments using DBSCAN with $\left( {\varepsilon ,n_{min} } \right)$ determined according to (**d**,**e**).

To determine individual cell clusters and classify cells with regard to their individual position within a cluster, i.e., being at the edge or surrounded by other cells, the density-based spatial clustering of applications with noise (DBSCAN) algorithm^11^ requires the specification of two parameters: the radius parameter $\varepsilon$, and the minimal cell number $n_{min}$ in a cluster. The algorithm examines the local density of cells within a radius $\varepsilon$ around each cell. Cells having at least $n_{min}$ number of cells within this radius are classified as *center* cells, while cells with fewer cells in the surrounding are specified as *edge* cells—when there is a center cell in their surrounding—or *noise* cells otherwise (Fig. 1b). The choice of the parameter combination $\left( {\varepsilon ,n_{min} } \right)$ has important implications for the classification of individual cells (Fig. 2a,b). For example, if for a given parameter $n_{min}$ the radius $\varepsilon$ is chosen quite small, a lot of cells are classified as noise cells although they might actually be connected to other cell clusters. Analogously, if for a given $n_{min}$ the radius $\varepsilon$ is chosen too large, individual clusters might remain undetected and too many cells might get classified as center cells, although they could be possibly better qualified as edge cells (Fig. 2b). To determine general relationships for the appropriate definition of $\left( {\varepsilon ,n_{min} } \right)$, we analyzed several images of cells grown at different densities by systematically testing different parameter combinations, and selected appropriate combinations by visual inspection (see “Materials and methods” section). We found that the choice of appropriate parameter combinations is related to the average local cell density within each image, $\Phi$, with the average local cell density defined by applying a regular grid to the observed image and dividing the total number of cells within the observed image by the covered area, i.e., the number of grid sites with at least one cell present (Fig. 2c). Based on the manually determined parameter combinations, the radius $\varepsilon$ and the minimum cell number $n_{min}$ were then specified as functions of the average local cell density, $\Phi$ (Fig. 2d,e; Table 1; “Materials and methods” section). We applied this newly developed method to determine appropriate parameter combinations for $\left( {\varepsilon ,n_{min} } \right)$ to cell culture experiments on H2B-turquoise cells grown at different densities. Visual inspection indicated a reasonable identification of individual cell clusters when using DBSCAN with these parameters (Fig. 2f), especially with regard to the classification of *noise* cells, i.e., cells not belonging to any other cell agglomeration. However, independent of the parameter combinations used, we generally observe that cell classification as defined by DBSCAN (Fig. 1b) tends to classify cells as center cells that are arguably at the edge of a cluster, i.e., not fitting with the common definition of an edge cell as being at the border of a cluster (Fig. 2b,f).

**Table 1:** Estimating DBSCAN parameters using functional relationships dependent on the cellular

Parameter	Best estimate (± SE)
Equation (1): radius, $\varepsilon \left( \Phi \right)$	
$\alpha$	$2.2 \times 10^{4}\, \left( { \pm 7.8 \times 10^{4} } \right)$
$\delta$	$6.35\, \left( { \pm 3.07} \right)$
c	$93.42\, \left( { \pm 2.21} \right)$
Equation (2): minimal number of cells, $n_{\min } \left( \Phi \right)$	
$\alpha$	$2.13\, \left( { \pm 0.25} \right)$
$\beta$	$1.33\, \left( { \pm 0.03} \right)$
$\kappa$	$15.70\, \left( { \pm 4.93} \right)$
$c$	$4.48\, \left( { \pm 0.15} \right)$

Estimates for the radius, $\varepsilon$, and the minimal number of cells, $n_{{{\text{min}}}}$, as functions of the average local cell density, $\Phi$, by fitting the relationships as defined in Eqs. (1) and (2) to 50 manually selected appropriate parameter combinations based on images of T84pMx1-mCherry H2B-turquoise cells grown at different densities (see also Fig. 2d,e). The best fit, as well as the estimated standard errors (SE) for each of the parameters are shown using python’s *lmfit* function (see “Materials and methods” section).

### An Edge-Correction algorithm improves cell characterization by DBSCAN

**Figure 3:**
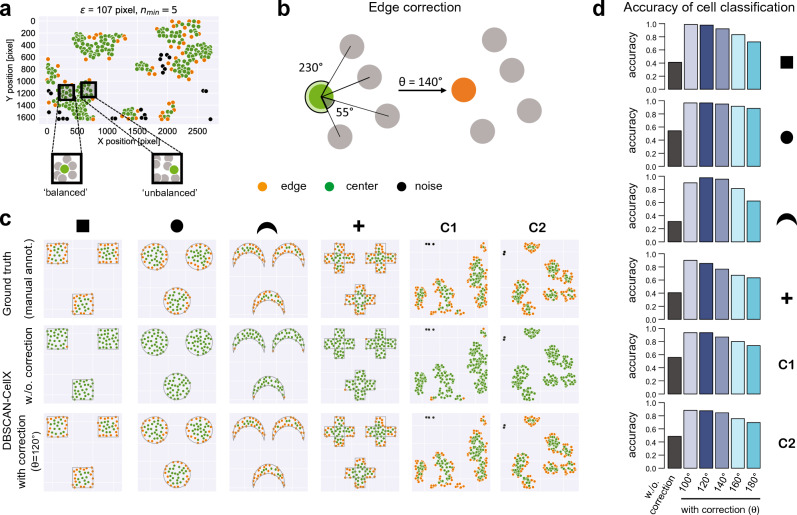
Edge-Correction algorithm: (**a**) Examples of cells classified as center cells by DBSCAN given more balanced and unbalanced distributions of the (at least) $n_{{{\text{min}}}}$ surrounding cells. (**b**) Schematic of the algorithm for correcting center cells as edge cells dependent on the angular distribution of neighboring cells by choosing an appropriate threshold value $\theta$ for the angular difference characterizing cell distributions as inbalanced. (**c**) Simulated data with cells distributed in varying regular shapes or based on Matérn Cluster processes using different parameterizations^16^. Classification of cells as edge and center cells is based on manually annotated ground truth (upper row), the standard DBSCAN-classification (middle row) and the novel edge-correction provided by DBSCAN-CellX using an angular threshold value of $\theta = 120^{^\circ }$ (lower row). For manually annotated ground truth cells were classified as edge cells if they have a direct connectivity to the border of the shape or cell cluster, respectively. (**d**) Accuracy of cell classification testing different values of $\theta$ (see also Fig. S1). Noise cells were not considered in calculation of the accuracy.

In order to improve the identification of cluster edges according to the common interpretation of cells as being at the border of a cluster, we developed an Edge-Correction algorithm as an extension to the standard DBSCAN classification method. The standard DBSCAN approach classifies a cell as a *center* cell if at least $n_{{{\text{min}}}}$ cells are within an area with radius $\varepsilon$ around the cell. However, this approach completely neglects the actual spatial distribution of the neighboring cells. If the distribution of the surrounding cells is unbalanced, i.e., if cells are only located in one specific local area around the cell, this cell might be better described as an edge cell (Fig. 3a). To determine the “balancedness” of the surrounding cells for each center cell, our newly developed algorithm evaluates the angle between each of the surrounding cells and a reference line (Fig. 3b). Sorting the angles and calculating the intermediate differences, a center cell will be re-classified as edge cell if one of these intermediate differences is larger than a predefined threshold value $\theta$ for the angular difference (Fig. 3b).

We tested our approach against several simulated data in which cells were randomly placed within regular and irregular shapes (squares, circles, etc.) (Fig. 3c). Cell classification by DBSCAN without and with our additional Edge-Correction algorithm was compared to manually annotated ground truth data, in which a cell is classified as an edge cell if there is a direct connectivity to the border of the shape, i.e., in line with the interpretation of edge cells as representing the smallest hull around a point pattern (Fig. 3c). For all tested shapes, the standard (i.e., uncorrected) cell classification by DBSCAN had a very low accuracy in identifying edge cells (Fig. 3d) reaching only values in between 0.44–0.56 across all simulated patterns. The Edge-Correction algorithm provided by DBSCAN-CellX outperformd the standard approach, showing accuracies between 0.76 and 0.98 dependent on the choice of $\theta$ and the underlying point pattern. Testing different threshold values, we found that best classification performance was achieved using threshold values between $\theta = 120^{^\circ } - 160^{^\circ }$, with results very robust when choosing $\theta$ within this range (Figs. 3d, S1).

**Figure 4:**
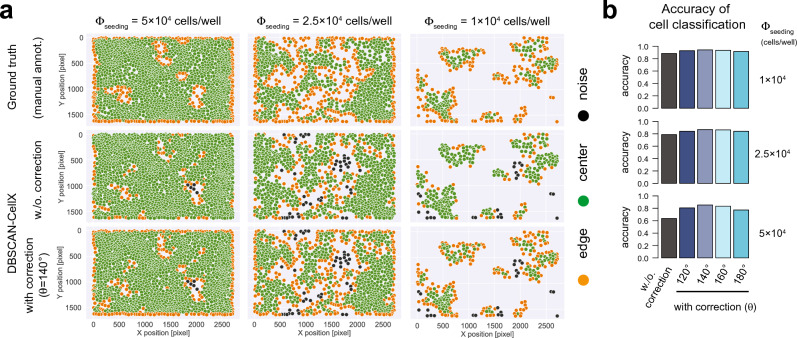
Edge-Correction improves cell classification in cell culture experiments: (**a**) Examples of cell characterization using standard DBSCAN methods (middle row) and the improved Edge-Correction algorithm (lower row) choosing a value of $\theta = 140^{^\circ }$ in comparison to manually annotated ground truth (upper row) based on images of T84 pMx1-mCherry H2B-turquoise cells grown at different cell seeding densities (compare to Fig. 2f). *Center* (green), *edge* (orange) and *noise* (black) cells are indicated as classified by the respective methods (see also Fig. S2). (**b**) Accuracy of cell classification testing different values of $\theta$ (see also Fig. S2). Noise cells were not considered in calculation of the accuracy.

We also applied our approach to actual experimental data of varying cell density (Fig. 4a). Also here we could see a substantially improved identification of edge cells by DBSCAN-CellX in comparison to the standard DBSCAN approach using manually annotated ground truth, with most appropriate results achieved for a threshold value of $\theta = 120^{^\circ } - 160^{^\circ }$ independent of the cellular density (Figs. 4b, S2). As the choice of $\theta$ might also depend on the specific cell type and cell density evaluated, the algorithm generally allows for user-defined threshold values.

### Determining cell embedding within clusters

Besides classifying cells as being at the edge or within a cluster, for analyses that aim to relate the functionality of a cell to its spatial positioning, it is also relevant to determine to which extent cells are embedded within a cluster, i.e., how distant they are to the cluster edge. As cluster edges represent access to the environment, this distance can have important implications for individual cell behavior with regard to the susceptibility to infection or cell reactivity. Therefore, we developed a method that assesses the distance of individual cells to the closest edge of the cluster that they belong to.

**Figure 5:**
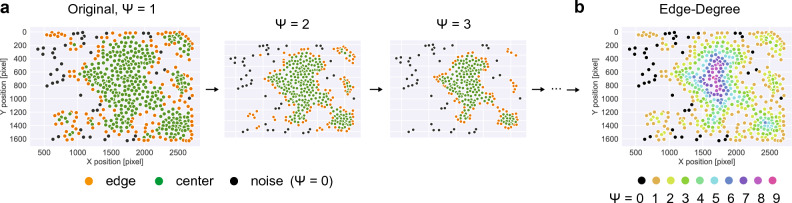
Edge-Degree specifies centrality of cells within clusters: (**a**) Representation of the stepwise-algorithm consisting of the combined application of DBSCAN with Edge-Correction to the data that are subsequently depleted of all classified *edge* cells before the evaluation is repeated on the reduced data set. (**b**) Higher edge degrees indicate a deeper embedding of cells within the cluster with initial *noise* cells having an edge degree of $\psi = 0$ and edges of clusters given by $\psi = 1$.

The stepwise algorithm relies on a repeated application of DBSCAN with Edge-Correction to the data. After each classification round, all *edge* cells are removed before the analysis is repeated on the reduced data set (Fig. 5). This provides each cell with an edge degree value $\psi$, which determines the iteration step at which the cell would be classified as an edge cell, and, thus, indicates the embedding of a cell within the cluster. Higher edge degrees indicate a deeper embedding of cells within the cluster with noise cells having an edge degree of $\psi = 0$ and edges of clusters defined by $\psi = 1$. Knowing the average diameter of the investigated cell type, the continuous distance of a cell to the cluster edge can be calculated based on the edge degree.

### DBSCAN-CellX, an extension to DBSCAN for the spatial analysis of cell cultures

In order to validate the general appropriateness of the developed methods for cell culture experiments, and the determined functional relationship for the DBSCAN parameters $\left( {\varepsilon ,n_{min} } \right)$ and the local density $\Phi$ (Fig. 2d,e), we applied our methods to data from different experimental conditions. Besides the analysis of T84 pMx1-mCherry H2B-turquoise cells grown at different densities as shown above (Fig. 2f), we also applied our methods to data from Huh7 cells that were infected with Dengue virus^17^ or hepatitis C virus^18^ (Fig. S3). By this, we were able to test our approach against cell populations that differed in their growth patterns, as well as variations in image resolution. Our analyses indicate that the determined parameterizations for DBSCAN allow reasonable identification of cellular clusters for different conditions, with the Edge-Correction algorithm providing an improved classification of cells with regard to their spatial orientation within clusters according to visual inspection (Fig. S3). Hereby, variations to the assumed threshold angle for edge-correction, as well as accounting for the average cell size used for calculating the average local cell density $\Phi$ (see “Materials and methods” section), were required to account for different cell sizes and cellular patterns (e.g. convex vs. concave shaped cluster edges corresponding to foci of cells or “holes” in tissues, respectively).

**Figure 6:**
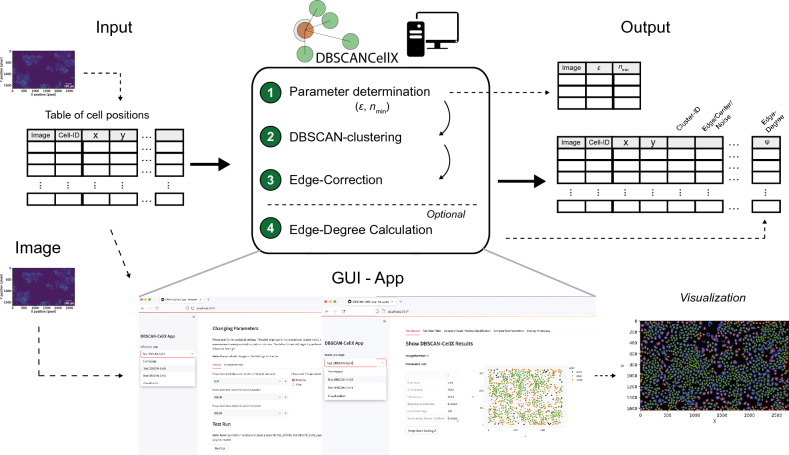
Schematic of the DBSCAN-CellX package: DBSCAN-CellX combines the original DBSCAN implementation with the extensions introduced above to allow an improved classification of cell clustering and individual cell positioning. DBSCAN-CellX performs (i) an automatic determination of appropriate parameter combinations of $\left( {\varepsilon ,n_{min} } \right)$ for DBSCAN based on cellular density, (ii) an Edge-Correction, and (iii) Edge-Degree determination. DBSCAN-CellX is developed as an OpenSource python-package and accompanied by an app which provides a user-friendly graphical user-interface that allows non-export users to perform cell classification and visual comparison of cell classification with the original images (see https://github.com/GrawLab/DBSCAN-CellX/ for further details).

We therefore combined the individual methods described above within the software package DBSCAN-CellX to allow an improved classification of the spatial distribution of cells and their relative location. Providing a table with the position of the individual cells as obtained by image analysis as an input, DBSCAN-CellX (i) automatically determines appropriate parameter combinations of $\left( {\varepsilon ,n_{min} } \right)$ for the application of DBSCAN, (ii) performs Edge-Correction according to the chosen angle $\theta$, and (iii) allows Edge-Degree determination. In addition, it includes several aspects that can also be optionally defined by the user (Fig. 6). We developed DBSCAN-CellX as an OpenSource python-package easy to use by experimentalists and non-expert users, and which is additionally accompanied by an app that provides a graphical user-interface, as well as visualization tools to qualify the analyses by overlaying original images with the obtained classifications. The package and additional documentation including its implementation is provided under https://github.com/GrawLab/DBSCAN-CellX/.

## Discussion

Revealing and characterizing the spatial distribution and organization of cells has become an important aspect for deciphering their interaction and functionality within tissues. Here, we developed novel extensions for the standard and widely used density-based clustering algorithm DBSCAN to provide an improved identification and classification of cells with regard to their spatial location.

One of the challenging steps for the application of DBSCAN is the appropriate determination of the parameters that define the assumed affiliation of a cell to a cluster, i.e., the assumed minimum number of cells $n_{min}$ within a radius $\varepsilon$ around each cell. With the outcome of the clustering analysis being sensitive to the choice of these parameters, we aimed for determining a functional relationship between the average local cell density within an analyzed image and the values of $n_{min}$ and $\varepsilon$ that provides appropriate parameter combinations for the application of DBSCAN to the data. Hereby, optimization especially focused on minimizing the determination of *noise* cells, i.e., cells not belonging to any cluster, as cells are usually assumed to grow within attached cellular agglomerates. We showed that DBSCAN-CellX provides appropriate cluster identification and spatial cell classifications regardless of the experimental dataset type (Figs. 2f, S3). However, the functional relationships to determine the parameters $n_{min}$ and $\varepsilon$ dependent on the average local cell density relied on simple phenotypic characterizations based on several independent observations. Additional analyses will be needed to determine if there is also theoretical evidence supporting the relationship between average cellular density and the appropriate choice of the parameters for DBSCAN, making the suggested relationship generally applicable. In addition, the parameter identification method within DBSCAN-CellX relies on the application of a regular grid to the image data to calculate average local cell densities. For our applications, we used a grid size of 20 μm which worked reasonably well for different experimental conditions. However, the appropriate choice of this grid size might depend on the actual cell sizes/diameters and image resolution, and more analyses are needed to analyze this point. Within the DBSCAN-CellX app, users are already able to account for this by varying the parameter influencing image resolution, and, thus, accounting for varying cell sizes.

By using DBSCAN, cell cluster identification by DBSCAN-CellX relies on this standard algorithm. With DBSCAN being generally sensitive to heterogeneous cell densities, several methods have already been developed to account for this issue. Extensions to DBSCAN that either use hierarchical clustering methods (HDBSCAN)^13^ or novel ordering methods of points regarding to their distances (OPTICS)^12^, allow the detection of clusters of different densities. However, despite reducing the number of dependencies, these algorithms still remain sensitive to the choice of certain parameters. Performing a rough comparison of DBSCAN with parameter identification provided by our method to HDBSCAN and OPTICS based on selected experimental data, we observe that our approach shows comparable performance to HDBSCAN with regard to cluster identification, as well as computational run time, with HDBSCAN seeming to be more sensitive to local density changes observable at the larger number of classified noise cells (Fig. S4). OPTICS seems to have problems with cluster detection for cells at high confluency, also needing a roughly 250–300-fold longer run time (Fig. S4). Thus, our parameter identification method could represent an appropriate and efficient extension for DBSCAN for identifying clusters within cellular monolayers.

Compared to HDBSCAN and OPTICS, the standard DBSCAN, and therefore also DBSCAN-CellX, additionally provides a classification of cells into *center*, *edge* and *noise* cells. DBSCAN-CellX was particularly developed to overcome arguable shortcomings of the edge-cell classification by the standard DBSCAN-definition (Figs. 1b, 2f). The developed Edge-Correction algorithm within DBSCAN-CellX, as well as the definition of the Edge-Degree developed herein, allow for a better characterization of cells dependent on their relative positioning within clusters, which has so far been insufficiently characterized by the standard DBSCAN-algorithm. This does not only apply to irregular patterns and cell densities as observed in cell culture experiments (Fig. 4) but even to regular shapes, in which the standard DBSCAN falls short in identifying edge cells according to the common definition, i.e., with edge cells representing the smallest hull around the identified cluster/point pattern (Fig. 3). While cell classification by the standard DBSCAN approach could potentially be improved by varying the parameters $\left( {\varepsilon ,n_{min} } \right)$ for a particular image, with results being sensitive to the choice of this parameter combination, the Edge-Correction algorithm provided here could be applied to any spatial point pattern that is obtained from a clustering method and seems to provide robust results for choices of $\theta \sim120^{^\circ } - 160^{^\circ }$.

Robust identification and characterization of cellular positions within tissues and multicellular systems have become important factors within different areas of biological research, such as organism development^19,20^, the functionality of heterogeneous microbial communities^21^ and individual cell physiology^22–25^. Moreover, recent advances in imaging and single-cell technologies, such as in-situ spatial transcriptomics, will increase the need for improved characterization methods of spatial relationships. In response to the increasing demands for analyzing the spatial distribution of cells and their response in tissue samples, several analytical methods and toolboxes have been developed recently, including PySpacell^26^, cytoNet^27^, CytoMAP^28^, and Context-Explorer^29^. While these tools provide sophisticated and advanced methods to determine cellular relationships directly accounting for functional and spatial connectivity, our method focuses on the classification of cellular positioning within clusters. Developed as an OpenSource python package that includes an App-based graphical-user interface, DBSCAN-CellX provides a simple tool that can be directly used by biological researchers to identify cellular clusters and determine the spatial organization of cells within them. By overcoming well-known shortcomings of the standard DBSCAN implementation specifically affecting the analysis of cell culture data or tissue monolayers, DBSCAN-CellX could help to decipher the influence of spatial context on cellular functionality within tissues.

## Materials and methods

### Software and algorithms

The DBSCAN-CellX package and all including analytical functions were developed in python using Python 3.8. DBSCAN-CellX can be run in a shell by bash-commands or via a local app containing a user-friendly graphical user interface within the local browser. The package including information concerning installation and system requirements, as well as a detailed documentation and a user manual can be found at: https://github.com/GrawLab/DBSCAN-CellX/

### Parameters of DBSCAN as functions of the average local cell density

To allow for an improved parameterization of the DBSCAN method, we assumed the following functional relationships between the parameters $\varepsilon$ and $n_{min}$, and the average local cell density $\Phi$: Based on the manually observed appropriate parameter combinations, we found that the radius $\varepsilon$ is best described by a function that assumes an exponential decrease with increasing average local cell density before reaching a saturation level. Thus, $\varepsilon$ is defined by

1 $$\varepsilon \left( \Phi \right) = \alpha e^{ - \delta \Phi } + c$$

with the parameter $\delta$ defining the rate of decrease, and $c$ the considered saturation level.

With $n_{min}$ only allowed to take discrete integer values, determining a functional relationship is more difficult. Given the determined parameter combinations, we assumed that $n_{min}$ is best described by a logistic function of the average local cell density $\Phi$:

2 $$n_{\min } \left( \Phi \right) = \frac{\alpha }{{1 + \left( {\frac{\beta }{\Phi }} \right)^{\kappa } }} + c$$

The actual values for $n_{min}$ are then obtained by rounding $n_{\min } \left( \Phi \right)$ to the next nearest integer.

For both functions, individual parameters were determined by fitting Eqs. (1) and (2) to the 50 manually determined appropriate parameter combinations using a maximum likelihood approach given in the package *lmfit* for python Version 1.0.3^30^. Individual parameter estimates and standard errors are shown in Table 1. Because $n_{min}$ is given as a discrete number when manually determining appropriate parameter combinations, for the fitting of Eq. (2) the data were weighted to ensure equal weights for each value of $n_{min}$ despite unequal numbers of manually determined parameter sets (compare Fig. 2e). Please note that both functions were developed based on the phenotypic shape observed within the experimental data, without particular functional evidence for these relationships. For the development of the functional relationships, the average local cell density was calculated using a regular grid with grid sites of $d = 20$ pixel according to the observed images. To account for varying cell sizes given different cell lines, for each specific experiment the optimal grid site is calculated based on the average cell size (diameter) $\eta$ provide in $\mu m$ and the image resolution $\tau$ given in $\mu m$ per pixel with $d = \eta /\tau$. Thereby, we additionally consider a scaling factor of $f_{base} = 1/\left( {\tau \times 2.8986\;{\text{px}}\;\upmu {\text{m}}^{ - 1} } \right)$ in Eq. (1) to scale for the image resolution of $\tau_{base} = 0.34\;\upmu {\text{m}}/{\text{px}}$ used within the experimental analysis for determining the parameters for Eqs. (1) and (2).

### Edge-Correction algorithm

As the standard DBSCAN algorithm tends to classify cells as center cells that are arguably at the edge of a cluster, i.e., representing cells that build the smallest hull around the cluster, the Edge-Correction algorithm tries to correct for this by determining the “balancedness” of the neighboring cells around a center cell (see also Fig. 3). To this end, each center cell $i$ is connected to all their $j = 1, \ldots ,m$ neighboring cells by an edge, $e_{ij}$, and the angle of each edge to a reference line, $\sphericalangle \left( {e_{ij} ,0} \right)$, is determined. The individual angles are then sorted and their intermediate differences are calculated $\left( {\sphericalangle \left( {e_{i2} ,0} \right) - \sphericalangle \left( {e_{i1} ,0} \right), \sphericalangle \left( {e_{i3} ,0} \right) - \sphericalangle \left( {e_{i2} ,0} \right), \ldots } \right)$ to determine, if there is at least one intersection that is larger than a certain threshold angle, $\theta$, which has been predetermined. If this is the case, the previously defined center cell is re-classified as an edge cell. The algorithm subsequently evaluates each center cell that has been identified by the standard DBSCAN method.

### Edge-Degree calculation

The edge-degree $\psi$ determines the embedding of cells within clusters. The edge degree is calculated by iteratively applying DBSCAN and the Edge-Correction algorithm to the data, in which in each step all edge and noise cells are removed before the algorithm is applied again (see also Fig. 5). The edge-degree value $\psi$ is an integer value that represents the step at which the corresponding cell would be classified as an edge cell, with original noise cells identified by $\psi = 0$, and original edge cells determined by $\psi = 1$. The higher $\psi$ the more central the cell is located within a cluster.

### Cell experiments

#### T84 pMx1-mCherry H2B-turquoise cells

WT T84 human colon carcinoma cells (ATCC CCL-248) expressing H2B-mTurquoise2 and pMx1-mCherry^31^ were maintained in a 50:50 mixture of Dulbecco’s modified Eagle’s medium (DMEM) and F-12 (Gibco) supplemented with 10% fetal bovine serum (FBS) and 1% penicillin–streptomycin (Gibco). Cells were seeded on plastic-bottom 96-well plates at 310,000, 230,000, 160,000, 80,000 and 31,000 cells/cm^2^. One day post-seeding, cells were fixed with 2% paraformaldehyde (PFA) for 20 min at room temperature. After PFA removal, cells were washed with 1 × PBS and permeabilized for 15 min with 0.5% Triton-X in PBS. To stain the nuclei, cells were washed with 1 × PBS and incubated with DAPI (4′,6′-diamidino-2-phenylindole) for 20 min. Cell imaging was done using the Widefield Celldiscoverer 7 (ZEISS) at a 5 × 2 magnification (Numerical Aperture = 0.35). The OpenSource software Ilastik 1.2.0 was used to generate a binary image representing each nucleus as an individual object in 2D based on the DAPI signal. Finally, using the OpenSource software CellProfiler 3.1.9, localization of each cell by x–y-coordinates was determined.

#### Huh7 cells exposed to hepatitis C virus

The experimental data and corresponding methods have been published in^18^. In brief, Huh7-Lunet CD81 MAVS-GFP-NLS cells were transfected with in vitro transcripts encoding the full-length HCV genome GLT1cc and co-seeded with naïve Huh7-Lunet CD81 MAVS-mCherry-NLS in a ratio of 1:5. After 72 h, cells were fixed with 4% PFA-PBS and stained with DAPI. Nuclear transfer of GFP or mCherry, respectively, identifies HCV positive cells due to cleavage of the MAVS-portion by the viral protease NS3-4A^32^, whereas uncleaved autofluorescent proteins are located at mitochondria in the cytoplasm.

#### Huh7 cells exposed to dengue virus

The experimental data have been originally published in^17^. 10^5^ cells were seed into a 35 mm-diameter glass-bottom culture dish (MatTek Corporation, USA) the day prior to infection. Cells were infected with dengue virus serotype 2 at an MOI of 10 TCID50 per cell for 1 h at 37 ºC with occasional rocking. After removal of the inoculum, cells were washed thrice with PBS and cells further grown in 2 mL imaging medium (phenol red-free DMEM supplemented with 100 U/ml penicillin, 100 mg/ml streptomycin and 10% fetal calf serum). Time-lapse microscopy was performed using a Nikon Eclipse Ti inverted microscope (Nikon, Japan) equipped with a motorized stage, climate chamber (EMBLEM, Heidelberg, Germany) and with a 20 × CFI Plan Apo lambda air objective (NA 0,75; Nikon, Japan). Twenty observation fields were manually selected, and 3-color images (bright field, GFP, TRITC (tetramethylrhodamine)) acquired at intervals of 30 min for 96 h using the automated Nikon perfect focus system.

### Supplementary Information

Supplementary Legends.

Supplementary Figure S1.

Supplementary Figure S2.

Supplementary Figure S3.

Supplementary Figure S4.

## Data Availability

All code is provided as an OpenSource software package including data examples under an MIT+-license following https://github.com/GrawLab/DBSCAN-CellX.
