# Supplementary material for: Extended methods for spatial cell classification with DBSCAN-CellX
Source: Sci Rep. 2023 Nov 1;13:18868. doi: 10.1038/s41598-023-45190-4 (PMC10620226; doi:10.1038/s41598-023-45190-4)
Supplement: Supplementary file 1 — Supplementary Legends. [file 41598_2023_45190_MOESM1_ESM.pdf]

Extended methods for spatial cell classification with DBSCAN-CellX

Supplementary Information - Figure Legends

(Numbers of references refer to the enumeration in the main manuscript)

Figure S1: Effect of the angular threshold θ on the re-classification of center cells given simulated data: Cell classification results for different values of θ for various examples of regular shapes used to test the performance of the Edge-Correction algorithm. Compare to Figure 3c,d in the main manuscript.

Figure S2: Effect of the angular threshold θ on the re-classification of center cells: Cell classification results for different values of θ for various examples of T84pMx1-mCherry H2B-turquoise cells grown at different cell seeding densities based on the data shown in Figure 4 in the main manuscript.

Figure S3: Application of DBSCAN-CellX to different experimental data: We used the determined functional relationship between the average local cell density Φ and (ε, n_{min}) developed based on T84 pMx1-mCherry H2B-turquoise cells to different cell culture experiments to tests its robustness. Application to cultures of Huh7 cells after being exposed to (**A**) hepatitis C virus [18] and (**B**) Dengue virus [17] showing application of DBSCAN-CellX to different conditions. Hereby, a threshold angle of $\theta = 140°$ (A), $75°$ (B, dense) and $140°$ (B, loose) was used for the Edge-Correction algorithm to allow for the detection of edge cells given various cell cluster shapes. For details on the generation of the experimental data see *Materials and Methods*.

Figure S4: Evaluation of DBSCAN-CellX against OPTICS and HDBSCAN: Determination of cell clusters based on HDBSCAN [13] and OPTICS [12] in comparison DBSCAN-CellX with (ε, n_{min}) as defined by the previously determined relationship (Eq. (1), Eq. (2)) for the latter. For HDBSCAN and OPTICS different values of the minimum cluster size, n_{min}, and minimum sample size, s_{min}, were tested and the best results are shown. Results are shown exemplary for different cell seeding densities of T84pMx1-mCherry H2B-turquoise cells. Used values always from top to bottom are (i) HDBSCAN $n_{min} = 6, 9, 6$; (ii) OPTICS $s_{min} = 6, 7, 10$; and (iii) DBSCAN-CellX $(\varepsilon, n_{min}) = (95,7), (101,5), (107,5)$. Computational run times show various performance between the algorithms, with HDBSCAN (0.07 s) showing equal performance to DBSCAN-CellX (0.04 s parameter determination + 0.02s clustering by DBSCAN), while OPTICS takes roughly ~250 to 300-fold longer (18.94 s). Run times are the average of 10 applications to different experimental data.
