# Supplementary figures and images for: Extended methods for spatial cell classification with DBSCAN-CellX
Source: Sci Rep. 2023 Nov 1;13:18868. doi: 10.1038/s41598-023-45190-4 (PMC10620226; doi:10.1038/s41598-023-45190-4)

# Ground truth

(manual annot.)

# DBSCAN-CellIX

w/o. correction

$\theta=160^\circ$

$\theta=140^\circ$

$\theta=120^\circ$

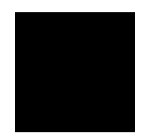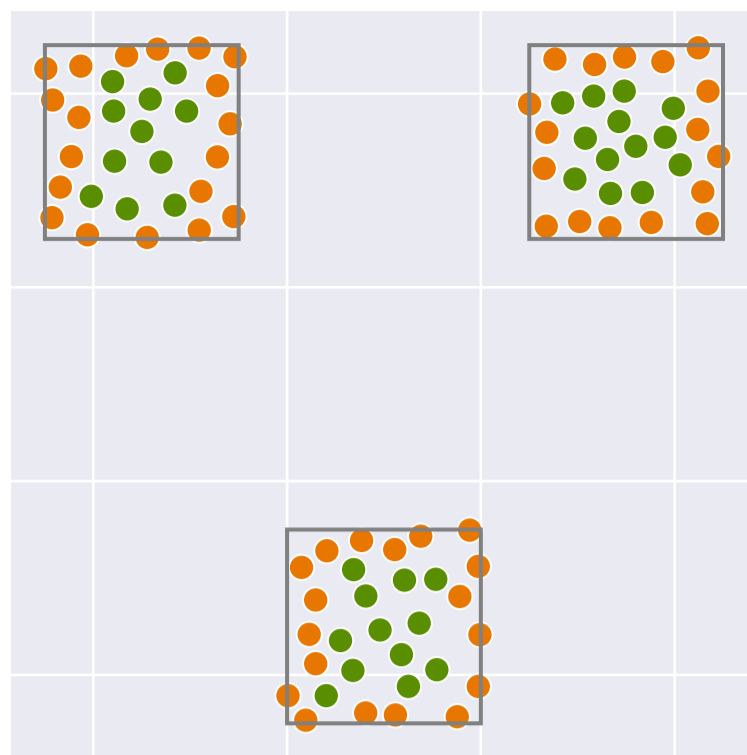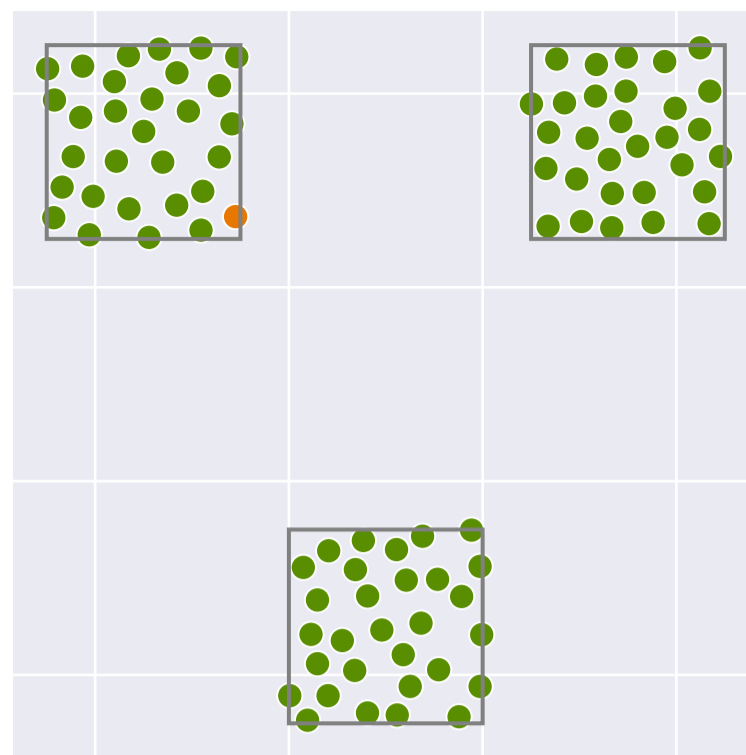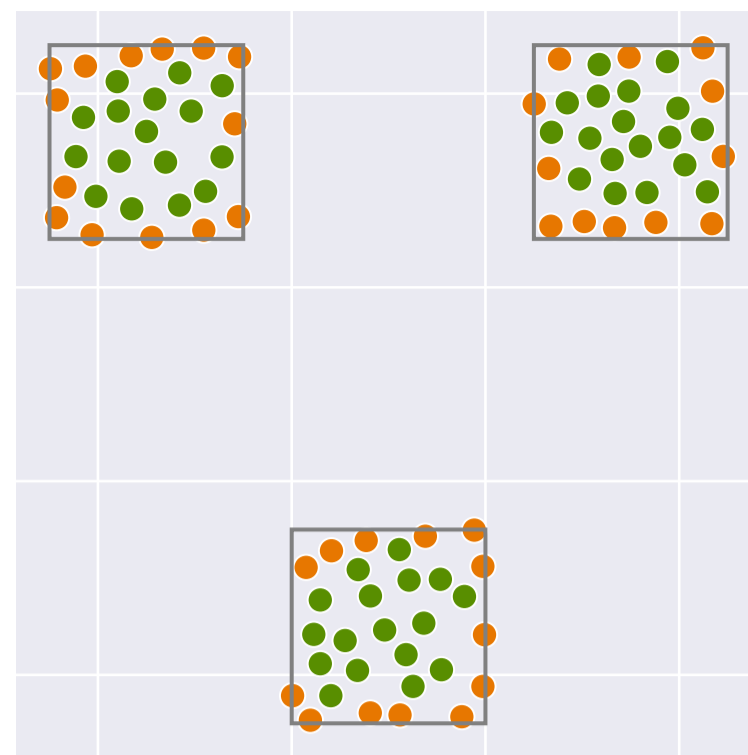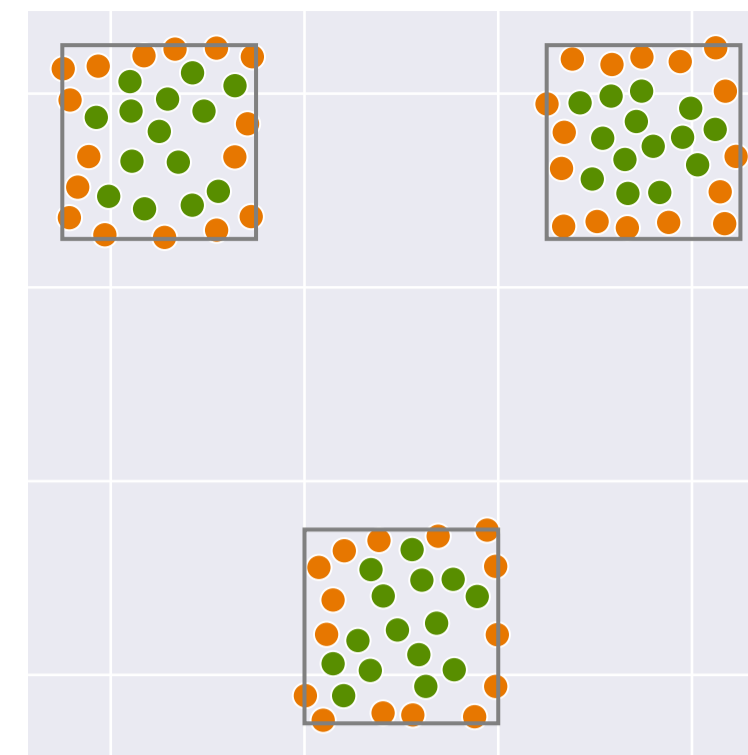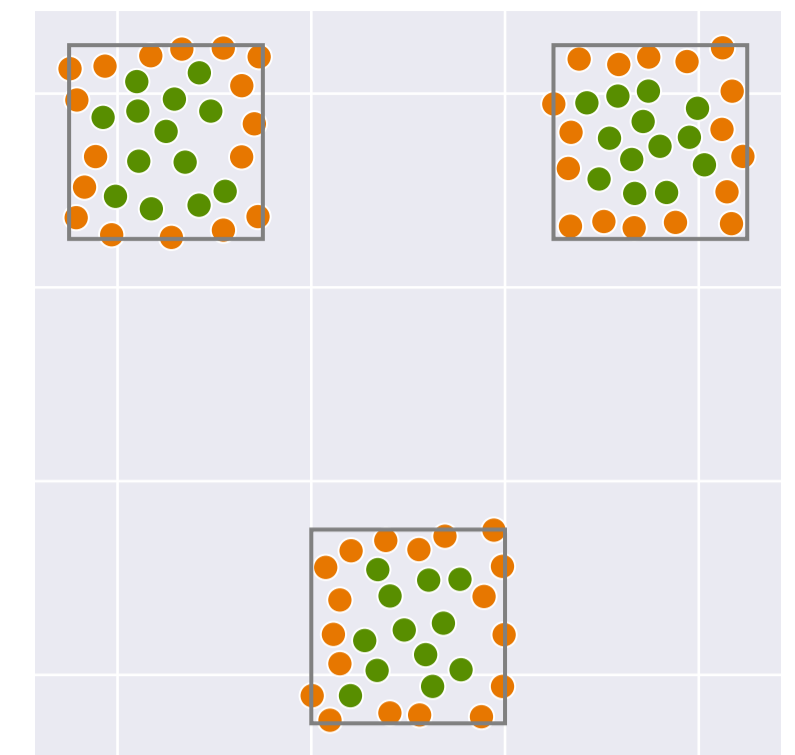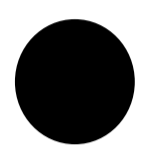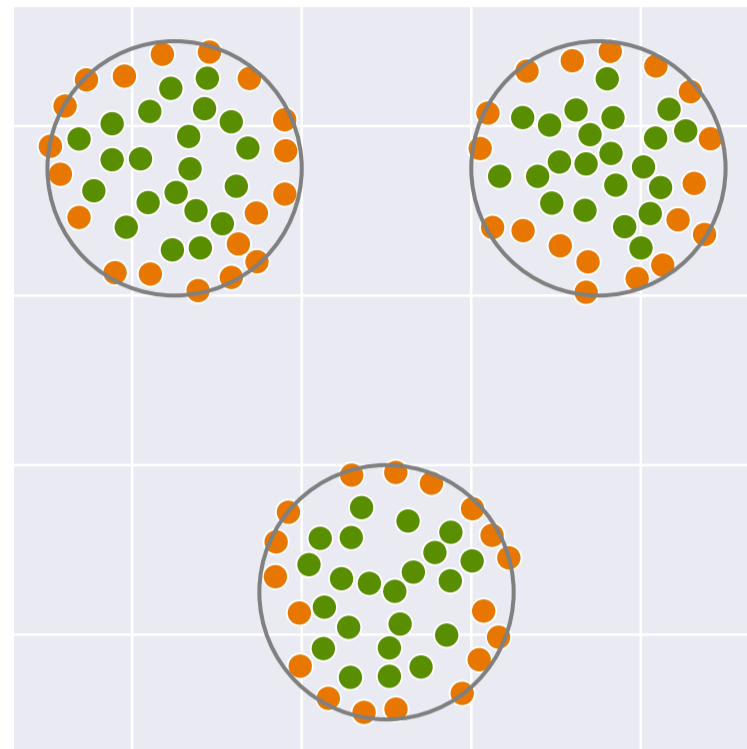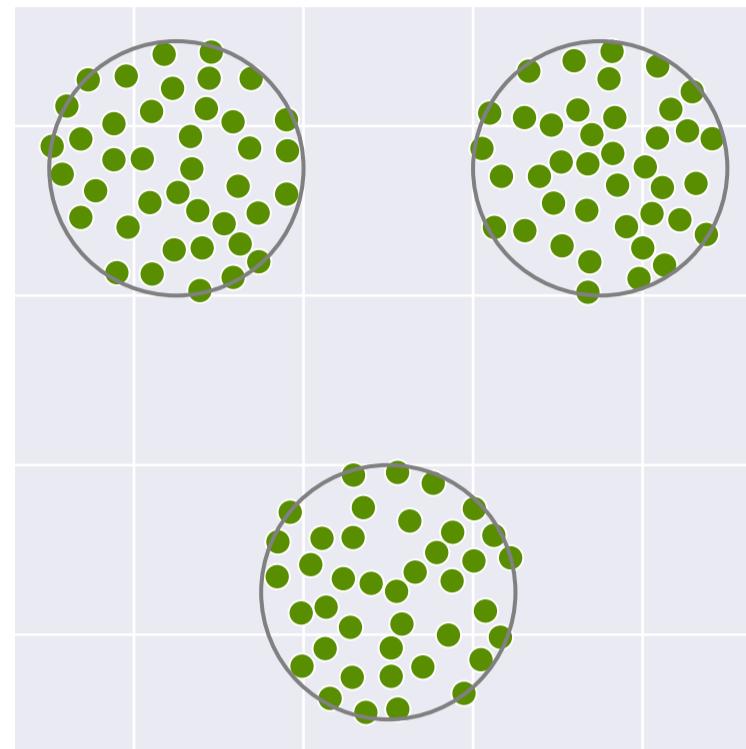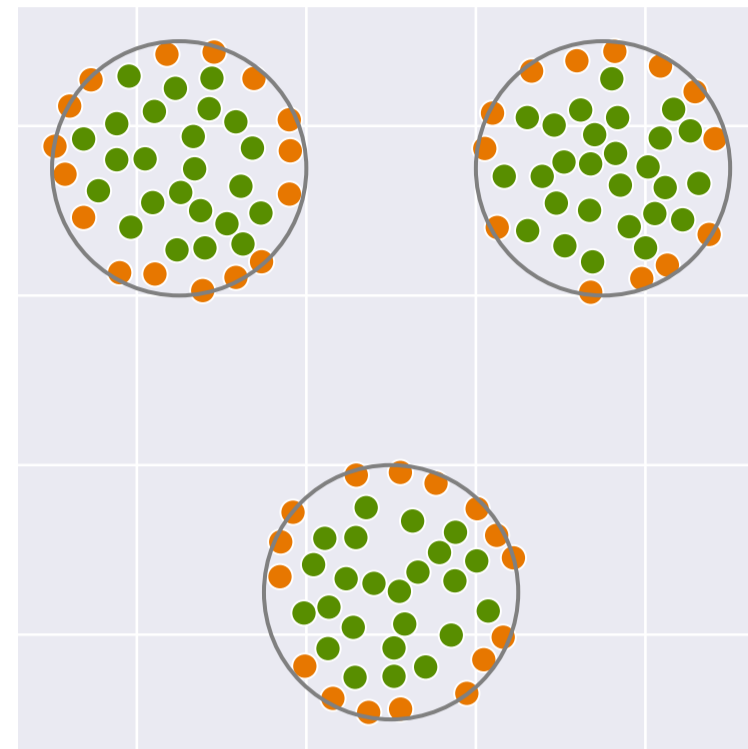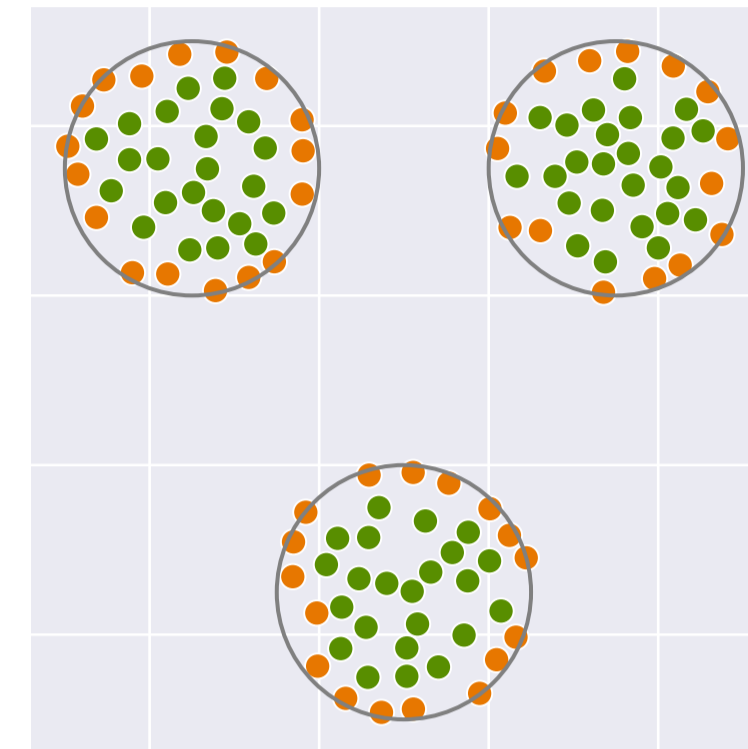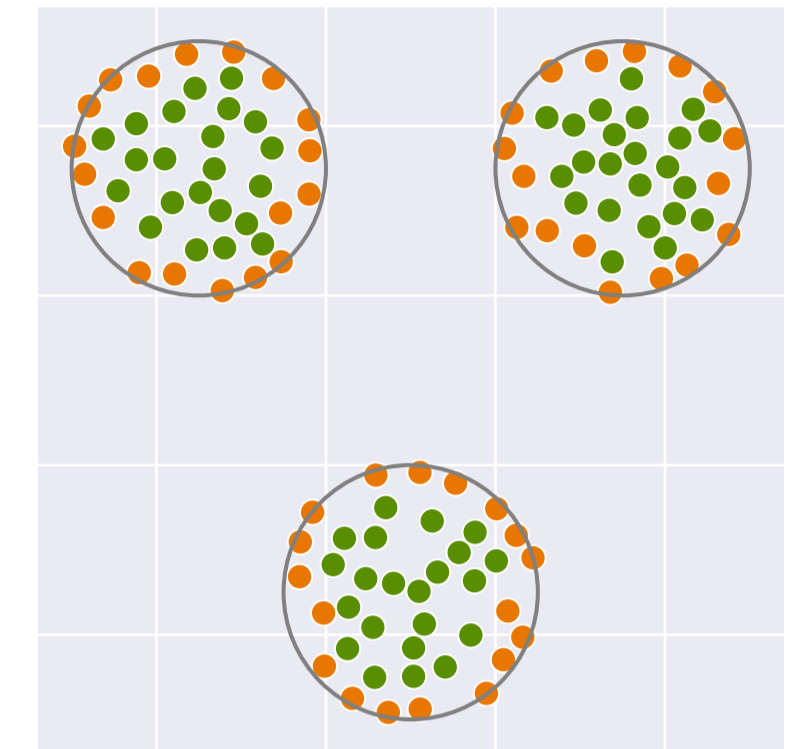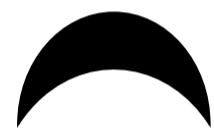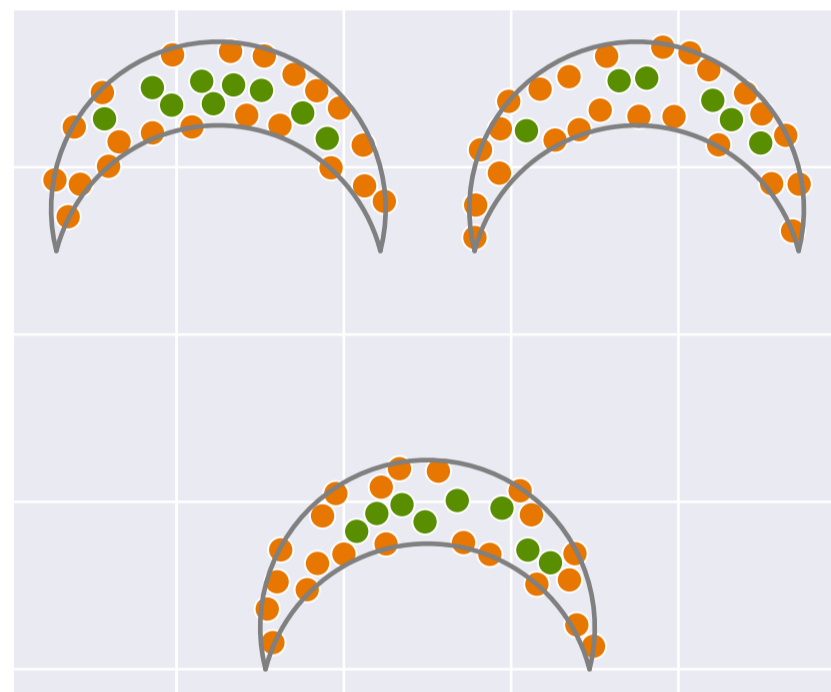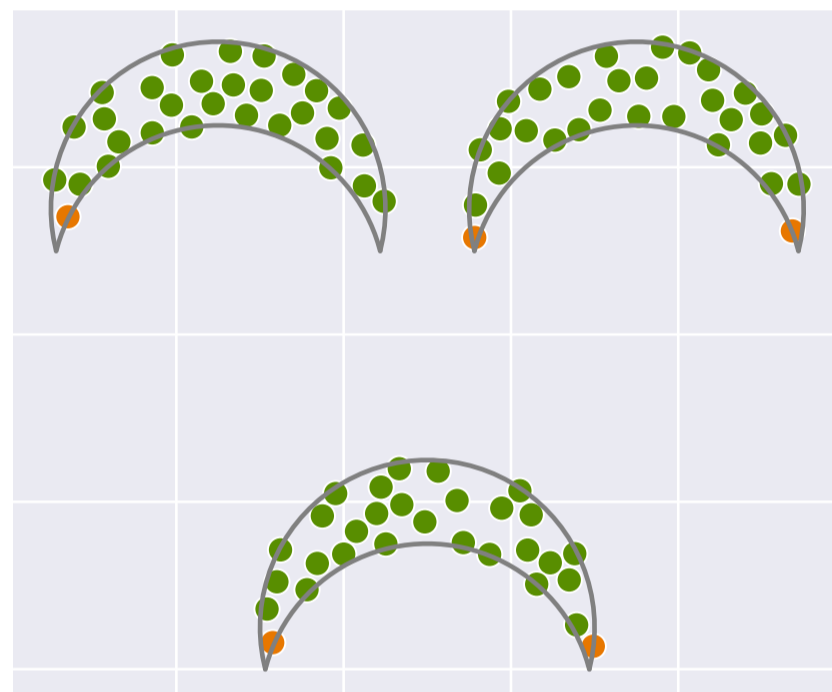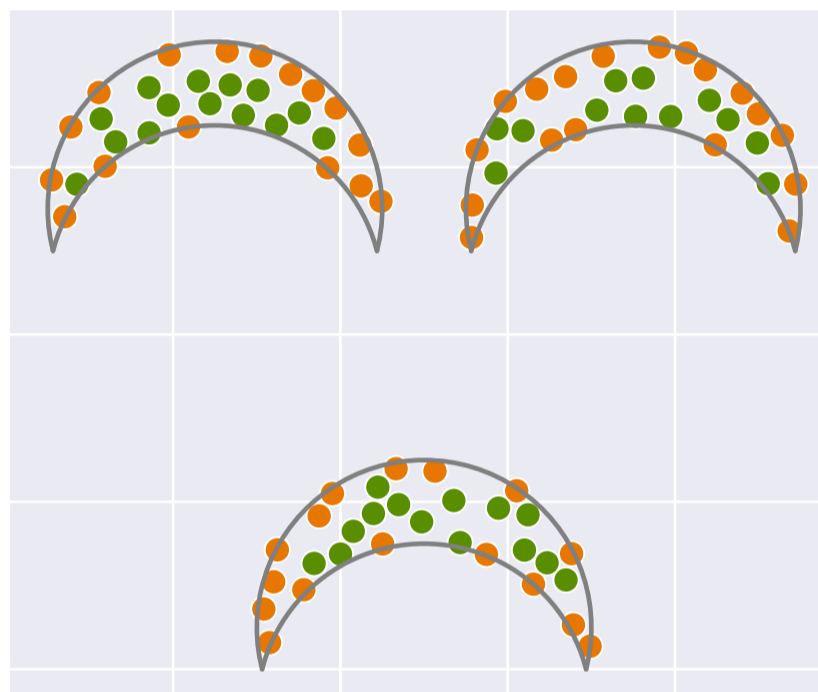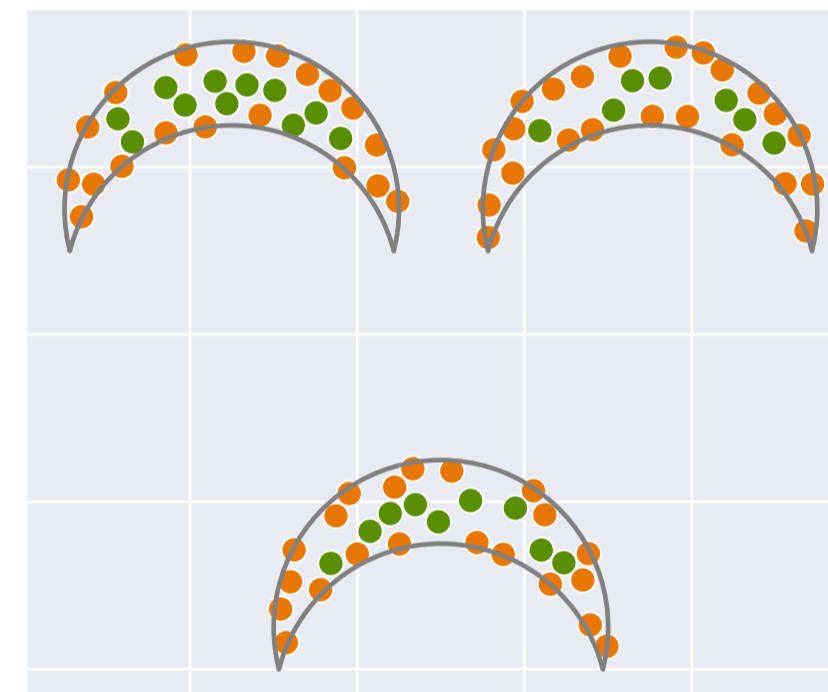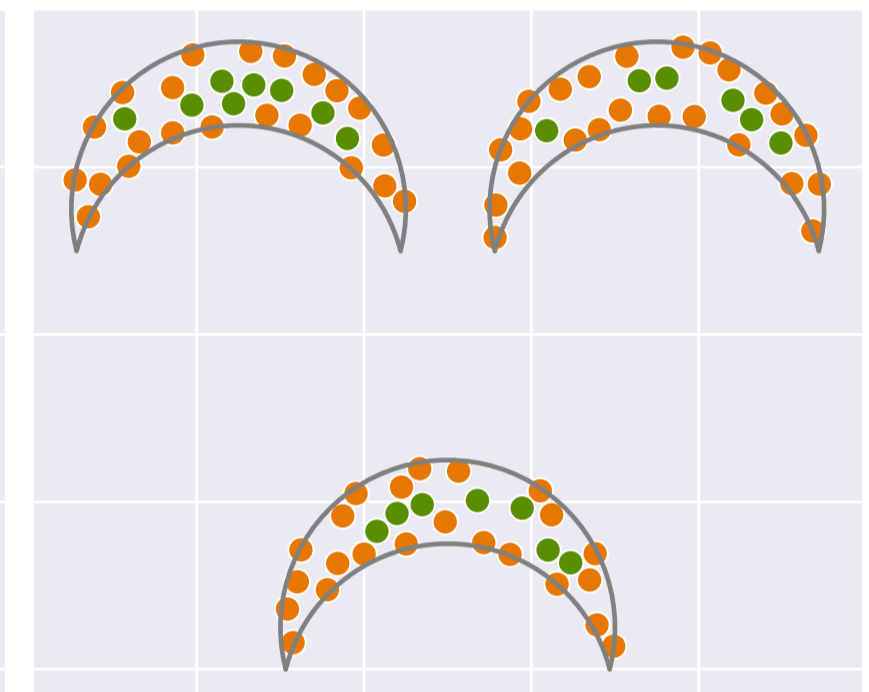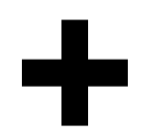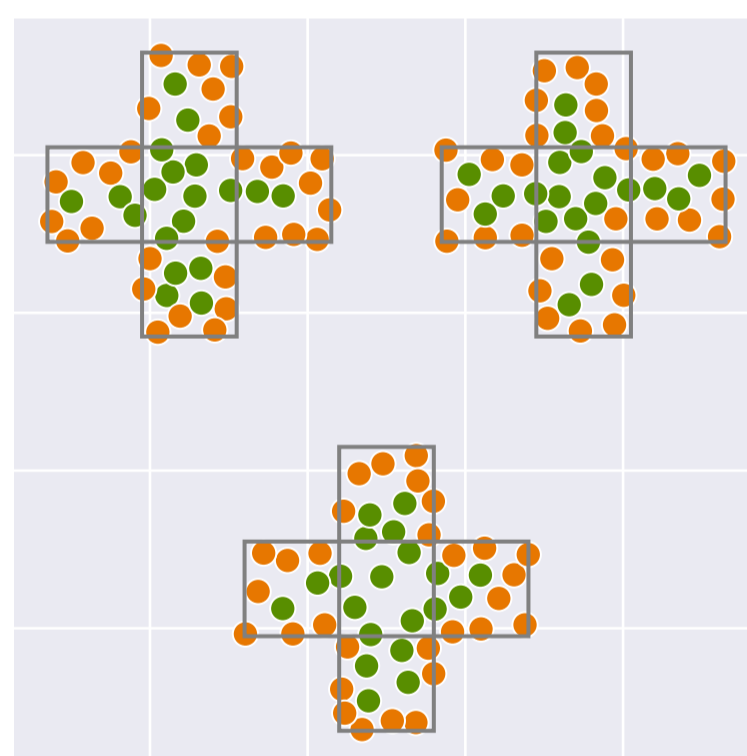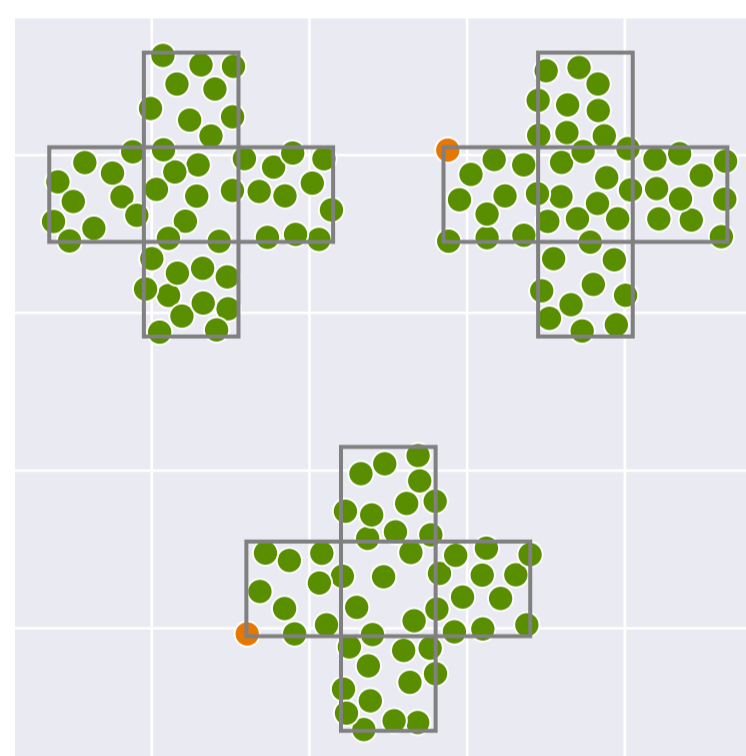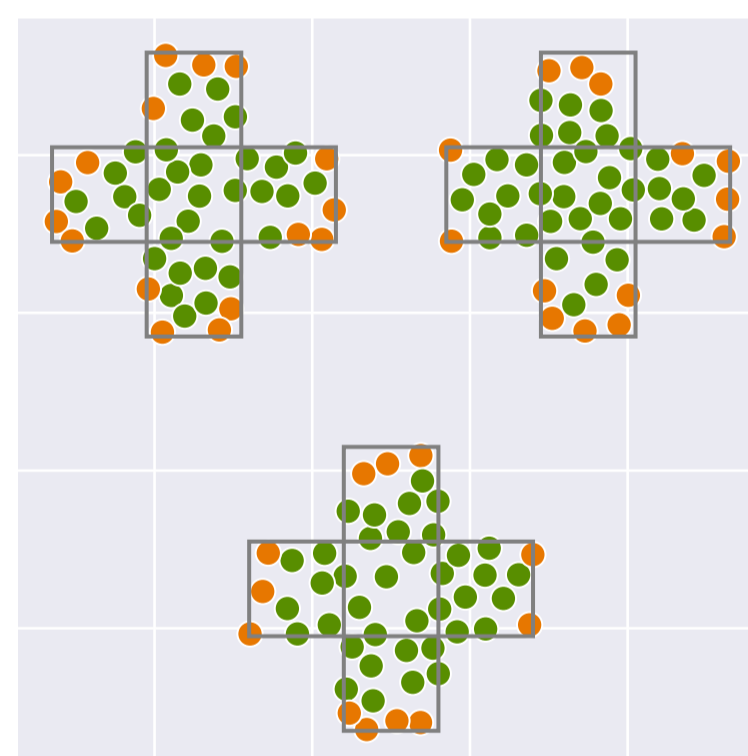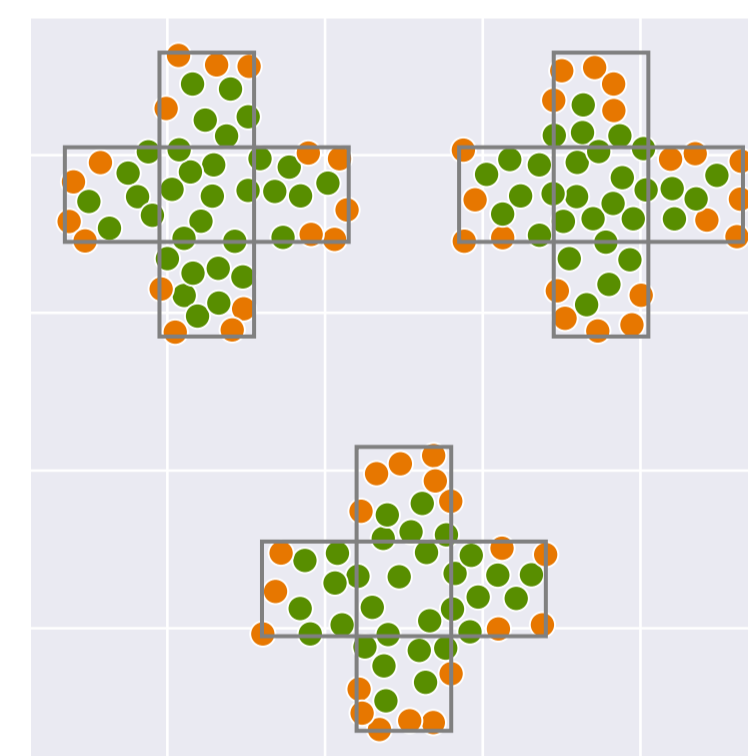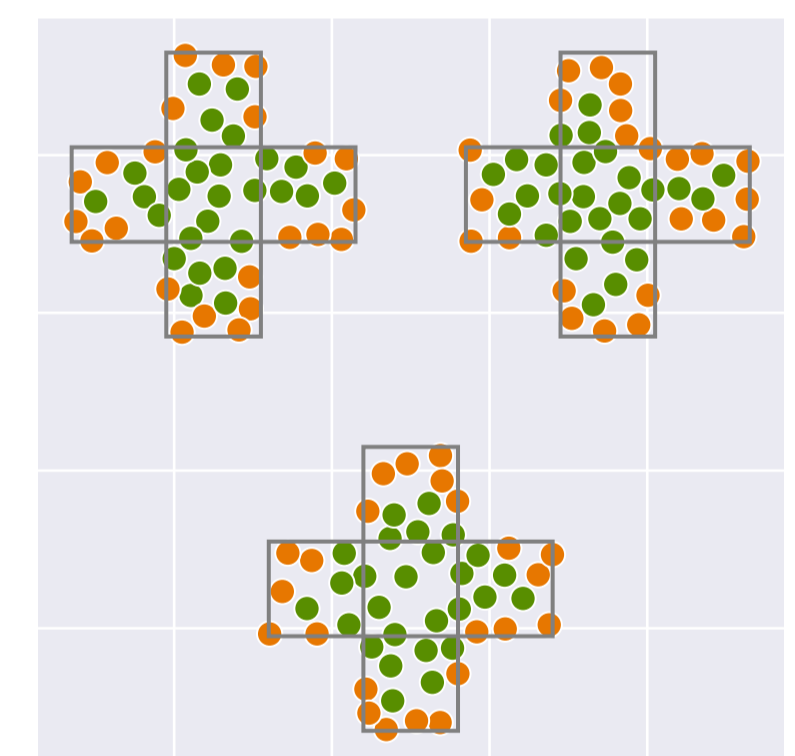

C1

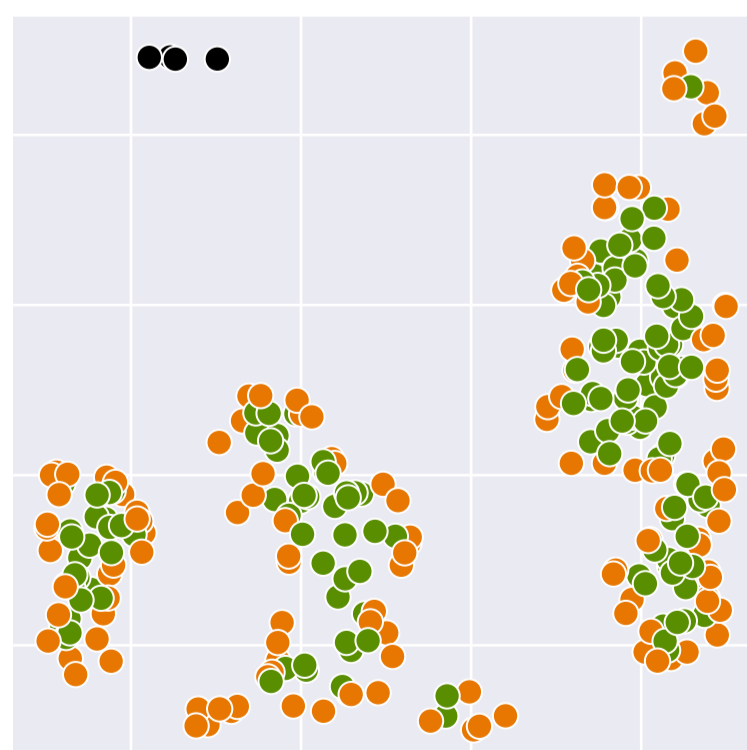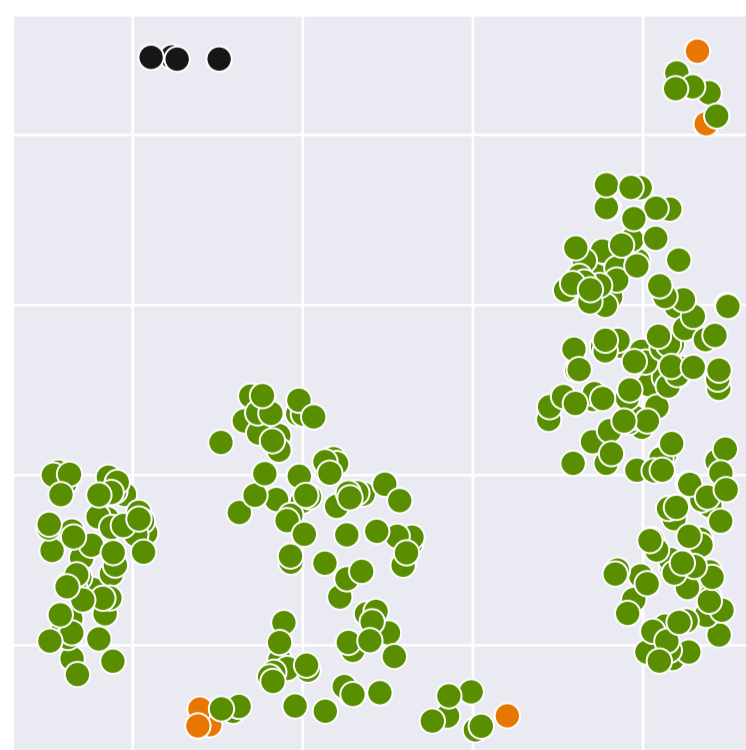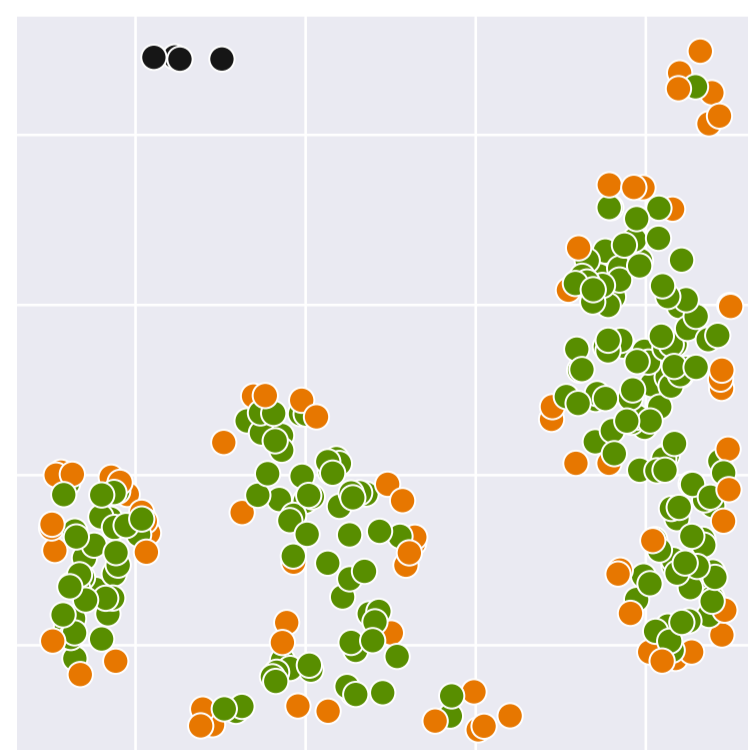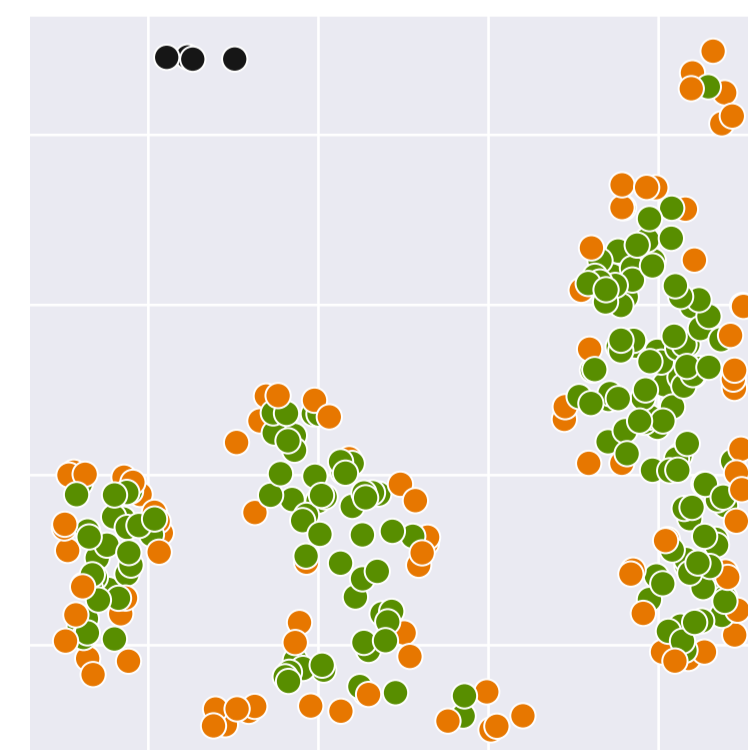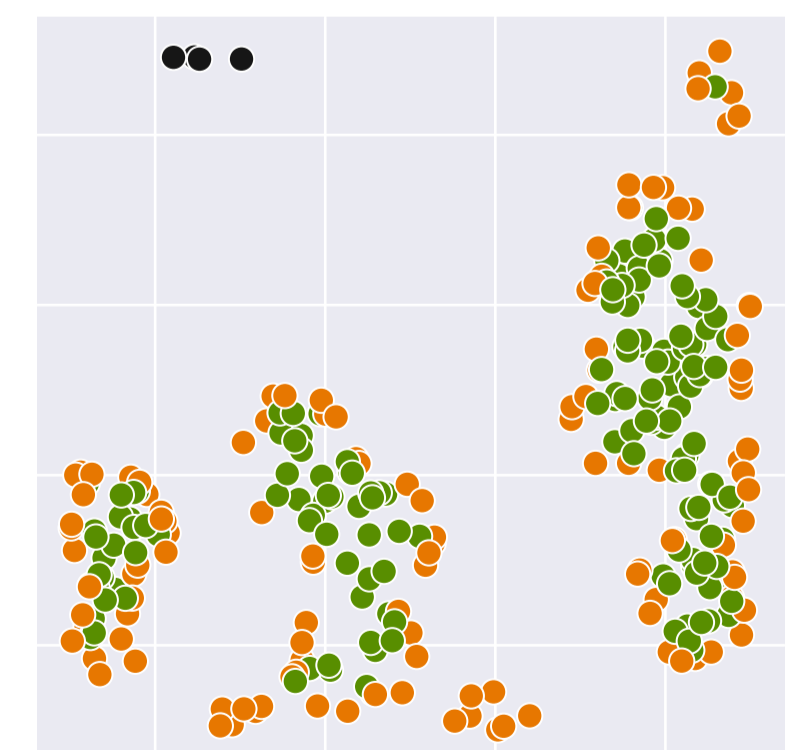

C2

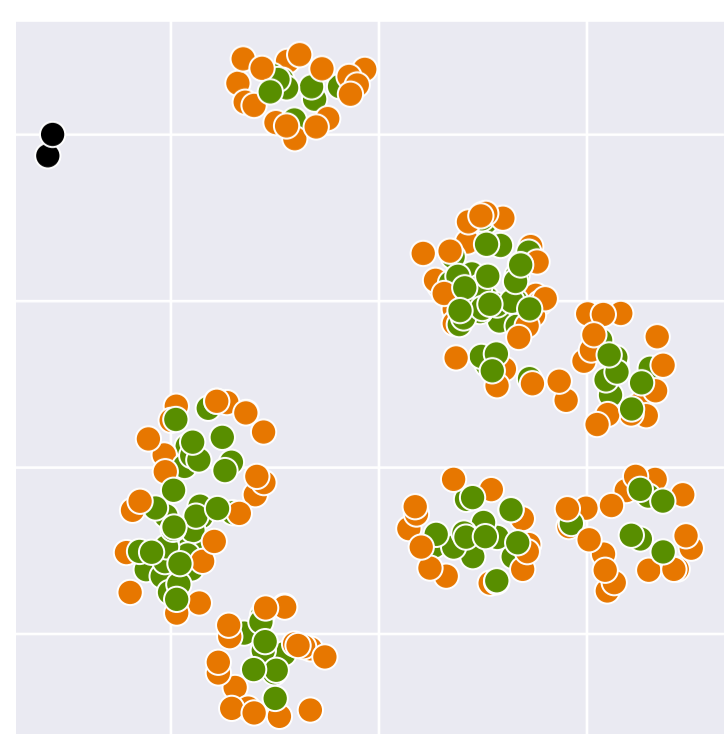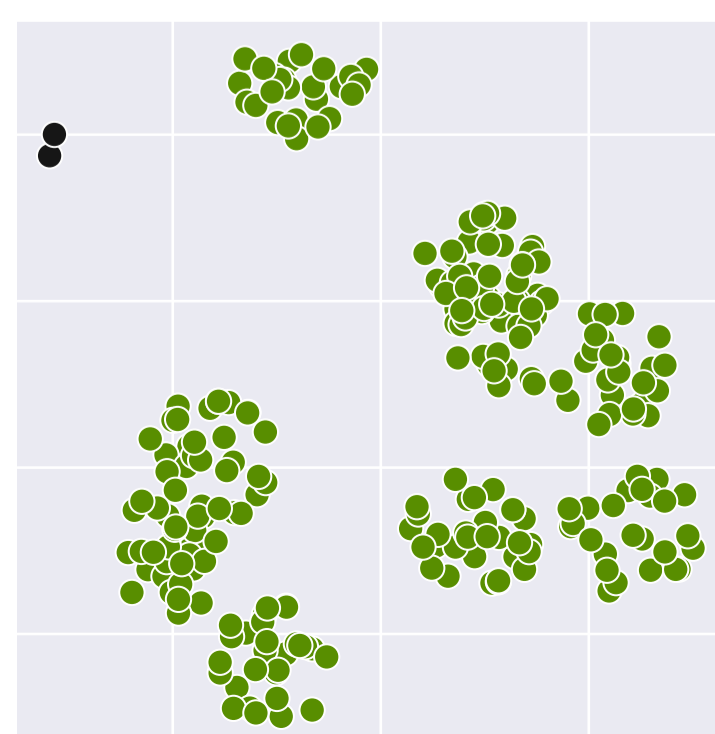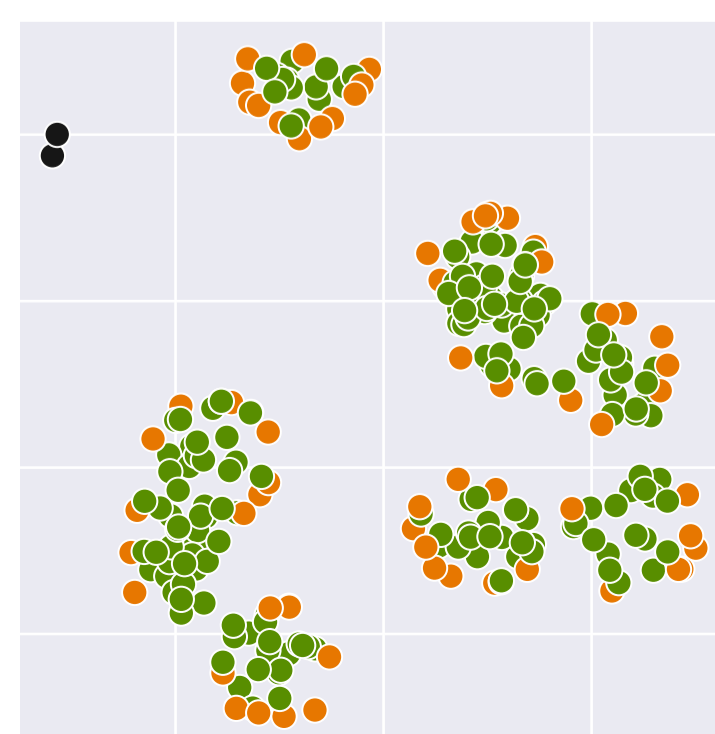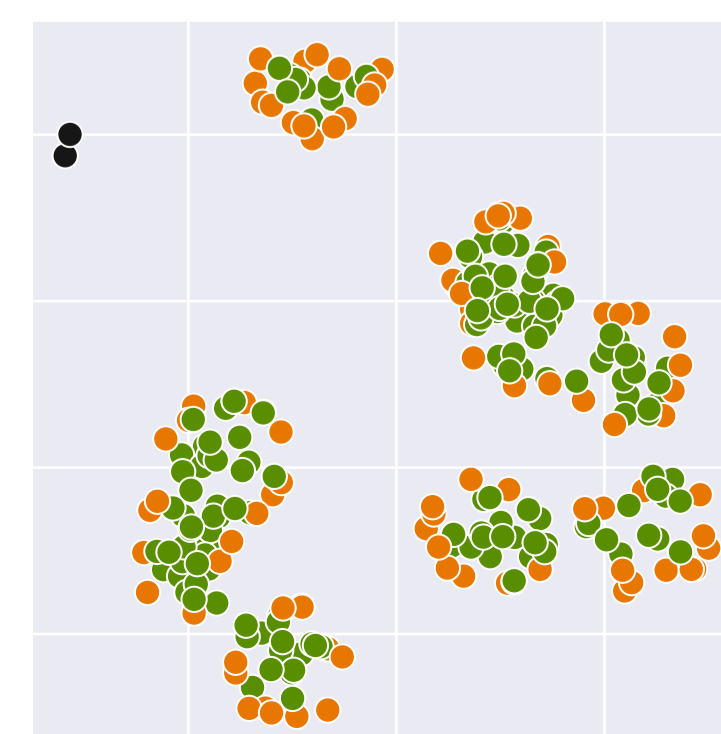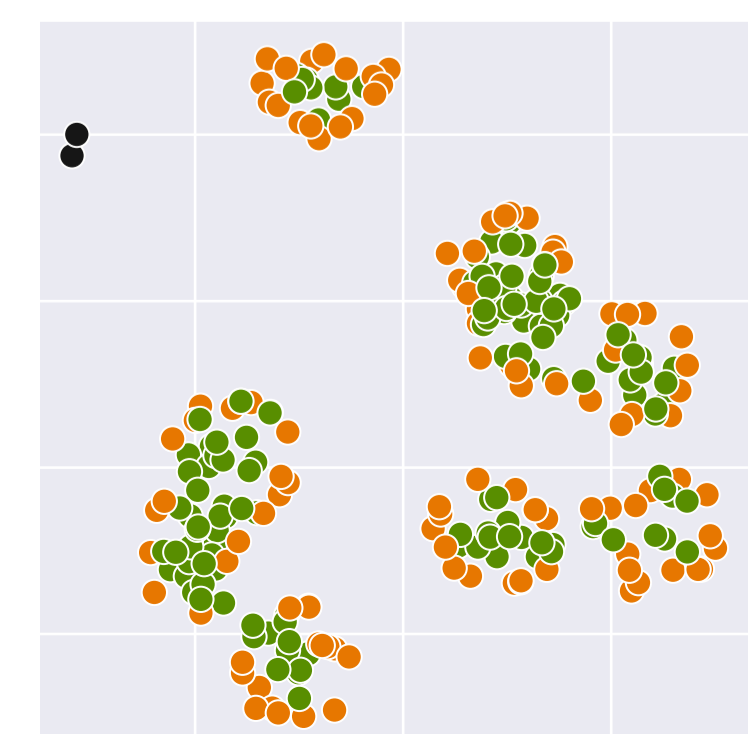

Supplement: Supplementary file 2 — Supplementary Figure S1. [file 41598_2023_45190_MOESM2_ESM.pdf]

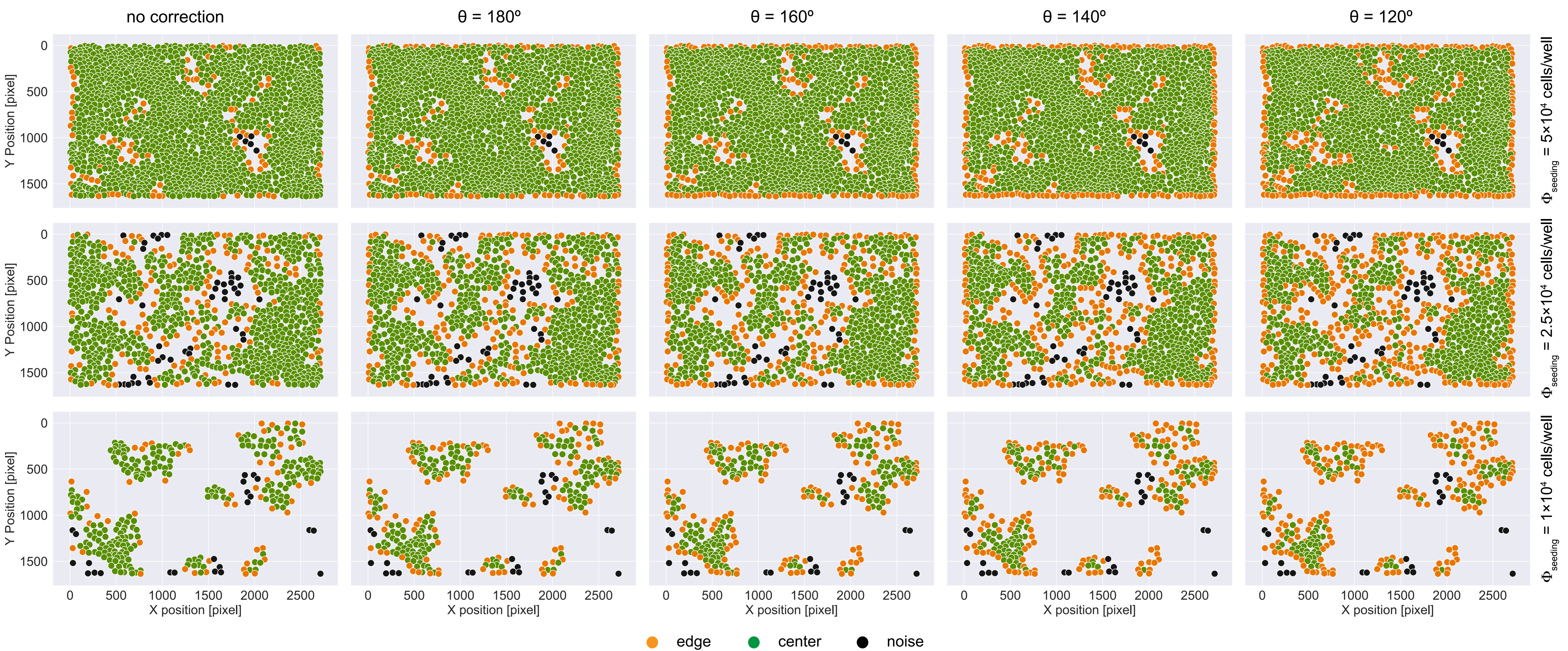

Supplement: Supplementary file 3 — Supplementary Figure S2. [file 41598_2023_45190_MOESM3_ESM.pdf]

HDBSCAN

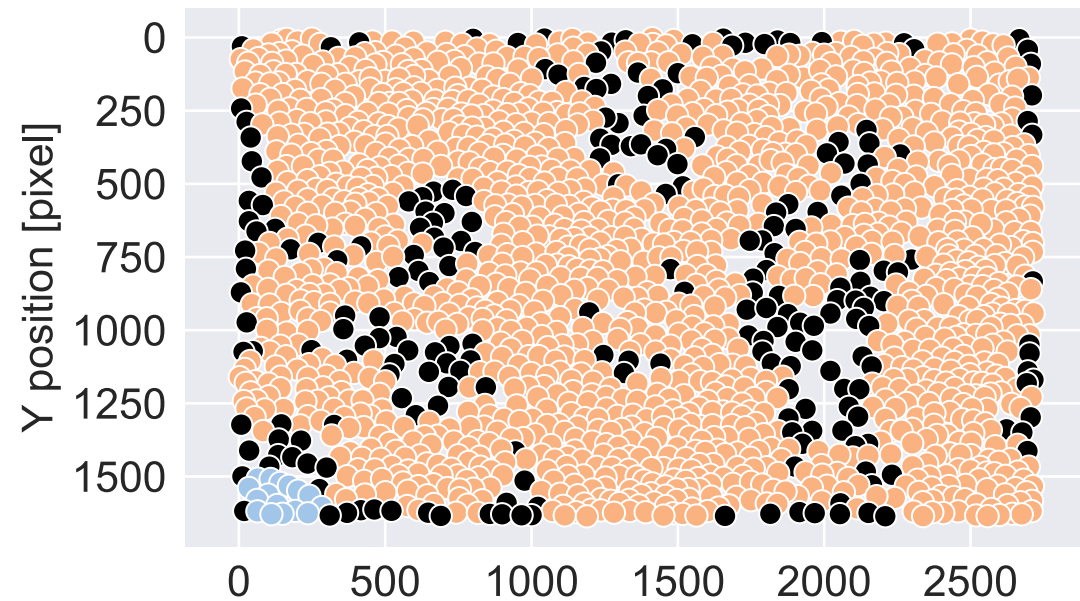

OPTICS

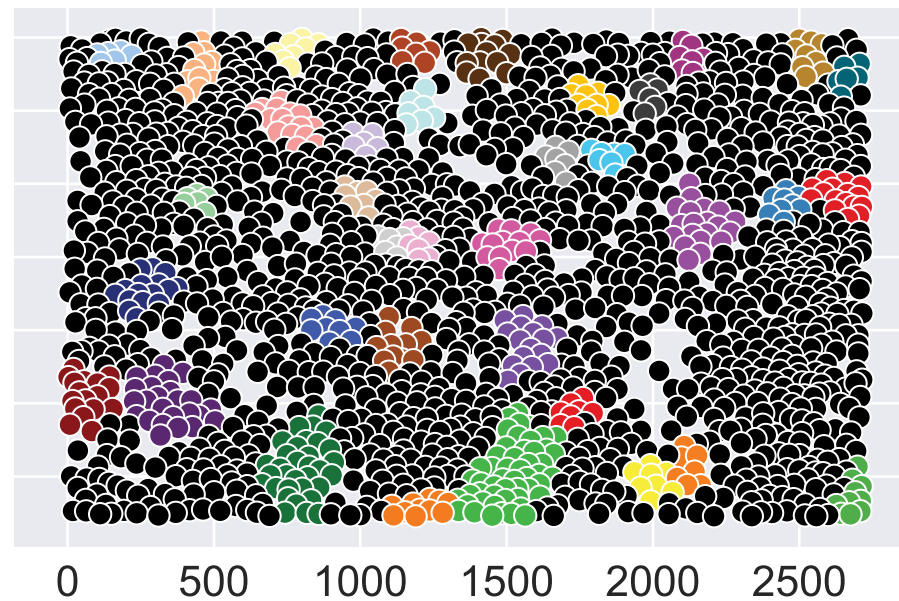

DBSCAN-CellIX

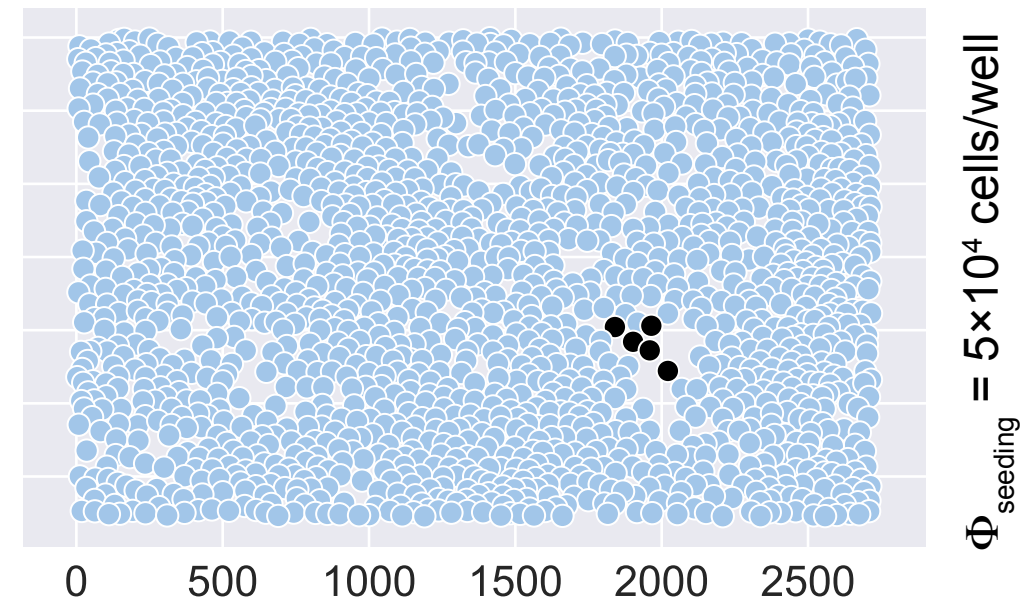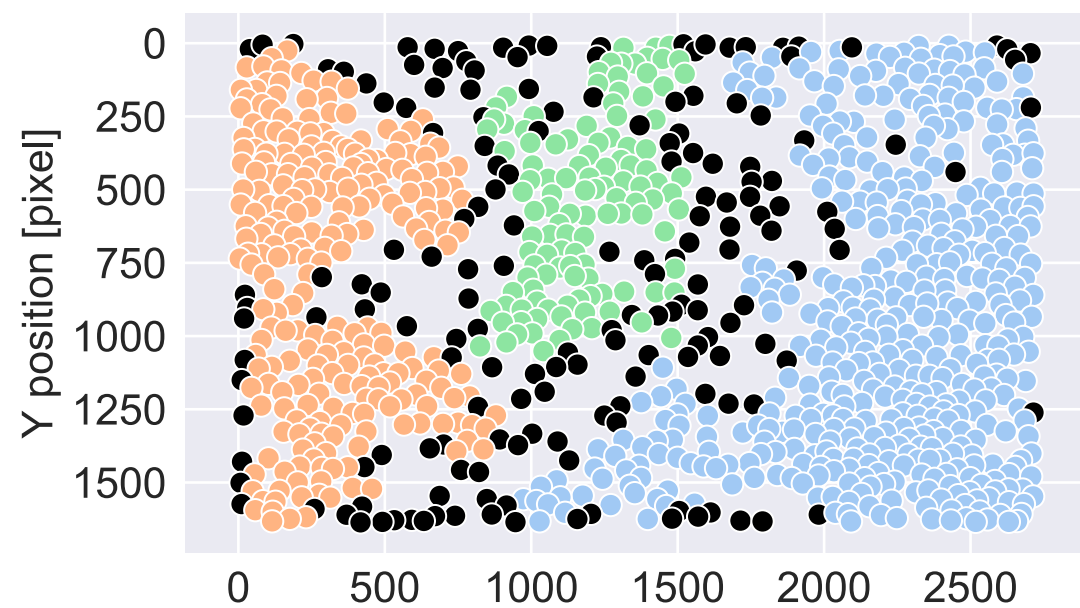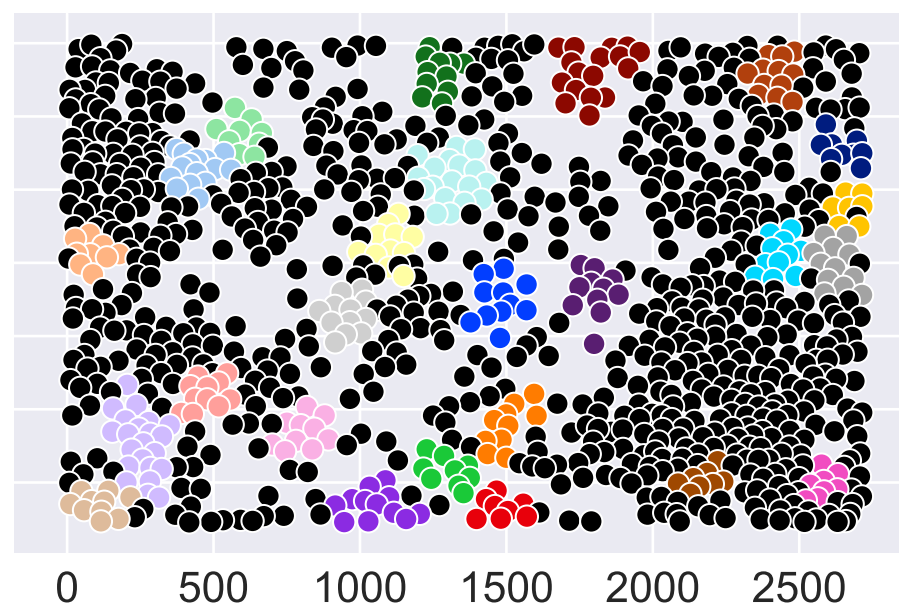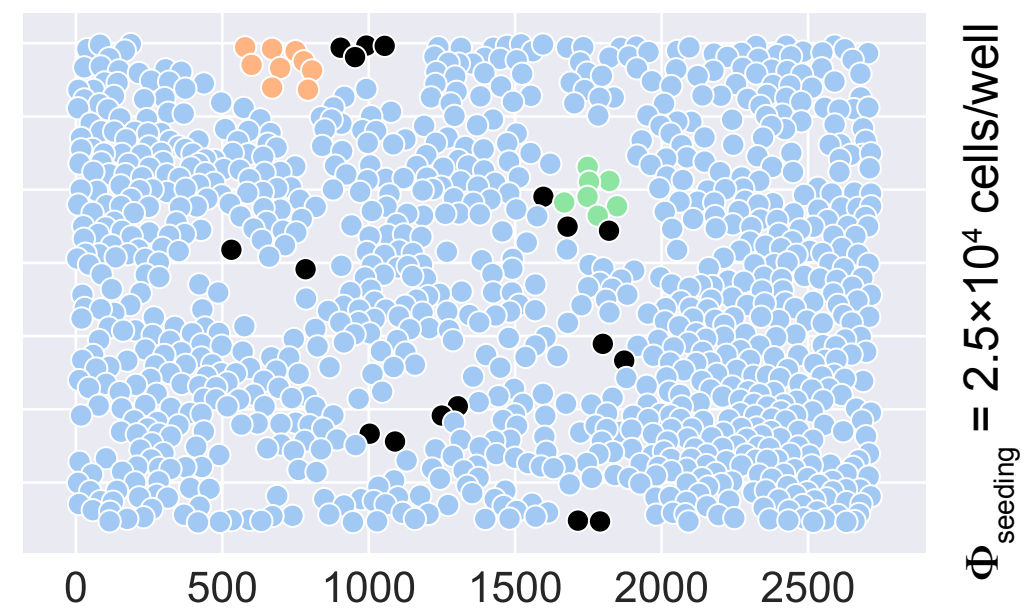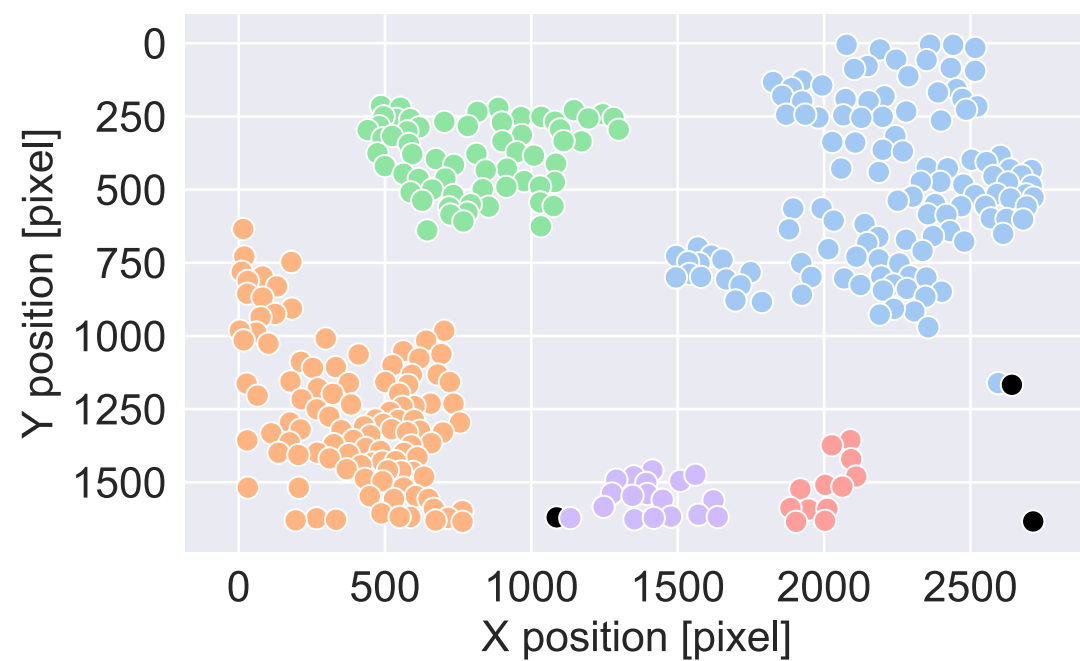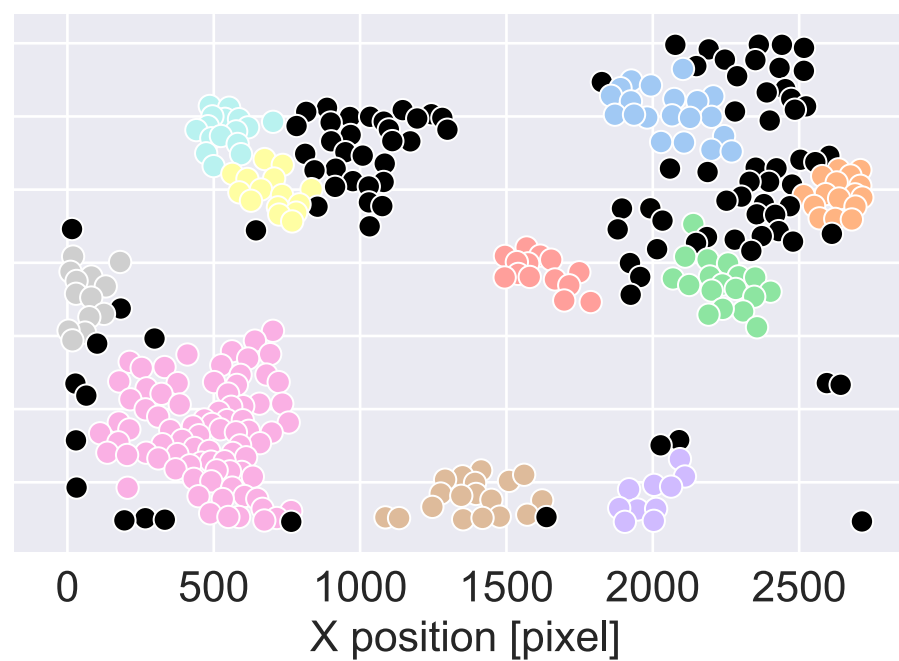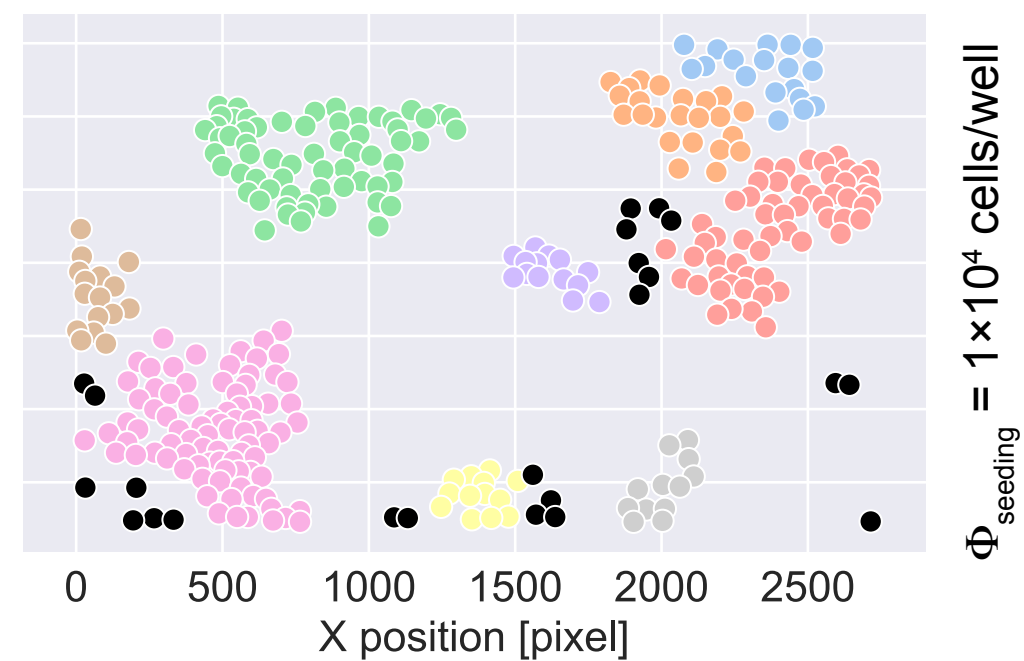

● noise      ● ● ● ● ● cluster

Supplement: Supplementary file 5 — Supplementary Figure S4. [file 41598_2023_45190_MOESM5_ESM.pdf]
