# Supplementary material for: Extended methods for spatial cell classification with DBSCAN-CellX
Source: Sci Rep. 2023 Nov 1;13:18868. doi: 10.1038/s41598-023-45190-4 (PMC10620226; doi:10.1038/s41598-023-45190-4)
Supplement: Supplementary file 4 — Supplementary Figure S3. [file 41598_2023_45190_MOESM4_ESM.pdf]

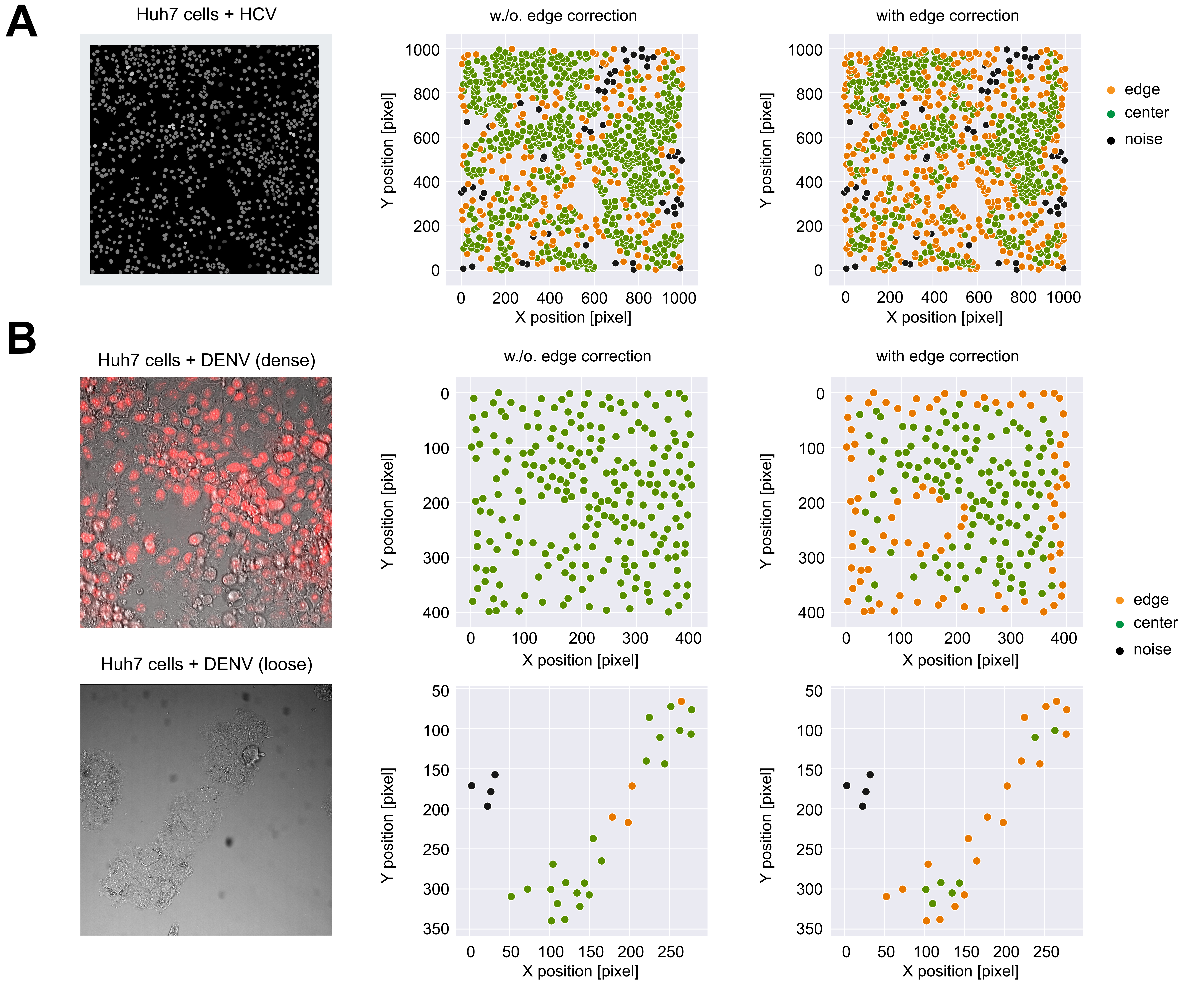

A
Huh7 cells + HCV
w./o. edge correction
with edge correction
Y position [pixel]
X position [pixel]
edge
center
noise
B
Huh7 cells + DENV (dense)
Huh7 cells + DENV (loose)
w./o. edge correction
with edge correction
Y position [pixel]
X position [pixel]
edge
center
noise
